# Integrated metabolomic and single-cell analyses reveal crotonylation-related epithelial remodeling and SDC4 dysregulation in adenomyosis

**DOI:** 10.3389/fnut.2026.1917718

**Published:** 2026-10-12

**Authors:** Jiaxi Wang, Yue Li, Hongyu Zhang, Yiling Zhao, Wei Fan, Wenhu Xin, Chunhua Qiang, Tiansheng Qin

**Affiliations:** 1 The Second Hospital and Clinical Medical School, Lanzhou University, Lanzhou, China; 2 Department of Gynecology, The Second Hospital and Clinical Medical School, Lanzhou University, Lanzhou, China; 3 Department of Obstetrics and Gynecology, People's Hospital of Tianzhu Tibetan Autonomous County, Gansu, China

**Keywords:** adenomyosis, fibrosis, lysine crotonylation, metabolic remodeling, NF-κB, syndecan-4

## Abstract

**Background:**

Adenomyosis (AM) is characterized by ectopic endometrial invasion and myometrial fibrosis, yet the molecular links between local metabolic remodeling, lysine crotonylation (Kcr), and epithelial dysfunction remain poorly understood. We investigated the metabolic and crotonylation-related landscape of AM and sought to identify epithelial molecular features associated with these alterations.

**Methods:**

Clinical metabolic profiles, public and newly generated tissue metabolomic data, crotonyl-CoA measurements, and tissue crotonylation assays were integrated with single-cell and bulk transcriptomic analyses. Kcr-related transcriptional patterns were characterized using multiple gene-set scoring approaches and co-expression analysis, followed by multi-algorithm feature prioritization. SDC4-associated epithelial states were further examined using trajectory inference, cell-cell communication analysis, transcription-factor prediction, and *in silico* perturbation.

**Results:**

Adjusted clinical analyses showed no robust differences in circulating lipid profiles, whereas tissue-level metabolomic analyses revealed alterations in lipid- and lysine-related metabolites in AM. Crotonyl-CoA estimates were higher in AM tissue, accompanied by increased H3K18cr. A higher p65-associated Kcr signal was also observed in a single IP comparison; given that only one IP measurement was available per group, this finding should be considered a preliminary descriptive observation. Single-cell analyses identified prominent Kcr-related transcriptional patterns in epithelial cells and linked this state to adhesion, junctional organization, and epithelial remodeling. SDC4 was consistently prioritized across five machine-learning procedures and was predominantly expressed in epithelial populations. SDC4-positive epithelial cells exhibited distinct inferred differentiation states and intercellular communication patterns, while computational perturbation further implicated SDC4-associated networks in epithelial adhesion, cytoskeletal organization, and junctional regulation.

**Conclusion:**

These findings connect local metabolic remodeling and crotonylation-related alterations with a distinct epithelial transcriptional state in AM and identify SDC4 as a consistently prioritized candidate associated with epithelial remodeling. The preliminary p65-associated Kcr observation provides additional context for investigating potential associations among Kcr, p65, and SDC4-related epithelial programs.

## Introduction

Adenomyosis (AM) is a common benign gynecological disease. Pathologically, AM is characterized by the presence of endometrial glands and stroma within the myometrium, a process thought to involve disruption or abnormality of the endometrial-myometrial interface (EMI). This process is accompanied by surrounding myometrial fibrosis and smooth muscle cell hypertrophy (1). The disease predominantly affects women of reproductive age. Reported incidence rates vary significantly, ranging from 5% to 70%, making it a frequently encountered condition in clinical practice (2). This ectopic histological distribution results in diffuse uterine enlargement and anatomical rearrangement. Furthermore, it is associated with prominent clinical manifestations, including progressively worsening secondary dysmenorrhea, menorrhagia, and chronic pelvic pain. These symptoms can substantially impair fertility and quality of life (3). Its impact on daily life and fertility resembles that of endometriosis, reflecting a burden where symptoms extend beyond the anatomical confines of the lesions (4). MRI helps delineate the extent of the lesions, though extreme caution should be exercised regarding the use of contrast agents during pregnancy (5, 6). Recent clinical evidence has also associated lower serum vitamin D levels with AM after covariate adjustment, underscoring the possible contribution of systemic host factors (7).

At the molecular level, the formation and progression of ectopic lesions involve complex pathophysiological processes. These processes include Epithelial-Mesenchymal Transition (EMT) and Extracellular Matrix (ECM) remodeling. They also involve increased protease secretion and the abnormal transformation of fibroblasts into myofibroblasts. Although the disease is biologically defined as benign, it exhibits invasive growth patterns and local infiltration characteristics similar to malignant tumors. Consequently, current hormone therapies or uterus-sparing surgical interventions often only alleviate clinical symptoms and are associated with high recurrence rates. Treatment decisions therefore need to balance symptom control and adverse effects against recurrence risk and fertility plans (8). Conservative medical and uterus-sparing surgical approaches do not provide a definitive cure. While hysterectomy serves as the definitive treatment for AM, it is unsuitable for patients who wish to preserve their fertility or their uterus (9). When definitive surgery is being considered, fertility counseling and, for selected patients, oocyte vitrification may be discussed before treatment (10–12). There is an urgent need for more precise therapeutic targets. This clinical dilemma stems from a lack of systematic understanding regarding the molecular regulatory networks. These networks drive endometrial cells to cross the interface and maintain their pathogenic phenotypes within the myometrial microenvironment. This need is especially relevant to individualized conservative management aimed at preserving fertility (13).

In recent years, the interaction between cellular metabolism and epigenetic regulation has attracted attention in reproductive disease. Related work has examined metabolic signaling, protein homeostasis, and chromatin regulation (14–16). A p300-related transcriptional complex has also been investigated in polycystic ovary syndrome (17). Lysine crotonylation (Kcr) is a non-acetyl acyl modification with a four-carbon unsaturated acyl group (18). Its chemical structure permits recognition by chromatin reader proteins and can influence transcription (19, 20). Intracellular crotonyl-CoA availability can affect p300-catalyzed histone crotonylation (21), while non-histone Kcr has also been implicated in metabolic and signaling functions. For example, ENO1 crotonylation has been associated with colorectal cancer cell phenotypes (22, 23). Crotonyl-CoA can be generated through lysine degradation, in which glutaryl-CoA is converted by glutaryl-CoA dehydrogenase, but this is not its only potential source (20). More generally, covalent-modification systems can amplify changes in substrate or enzyme availability (24). These observations provide a rationale for examining metabolite abundance and tissue Kcr together without assuming that one expression-defined pathway determines the crotonyl-CoA pool in AM.

The complexity of AM tissues is reflected in both pathological structure and high cellular heterogeneity. The lesions contain ectopic epithelial cells, stromal cells, infiltrating immune cells, and endothelial cells. Traditional bulk transcriptome sequencing can mask signals from relatively rare epithelial subpopulations with potentially important pathogenic roles (25). Metabolic remodeling and protein modifications exhibit significant cell-type specificity. The value of cell-specific analysis is also evident in human granulosa-cell studies of membrane transport and follicular-fluid signaling (26, 27). Therefore, analyzing tissue maps at single-cell resolution using Single-Cell RNA Sequencing (scRNA-seq) provides an important approach for identifying core pathogenic targets.

This study combined clinical reanalysis, public and newly measured tissue metabolites, and a crotonyl-CoA immunoassay with single-cell transcriptomics to investigate this possibility. Five machine-learning algorithms were used for exploratory gene prioritization, with SDC4 and WFDC2 tracked as biological candidates during reanalysis. SDC4 is a transmembrane heparan sulfate proteoglycan involved in cytoskeletal organization, adhesion, and migration; WFDC2 is a secreted glycoprotein studied in endometrial and ovarian disease contexts (28, 29). We prioritized SDC4 for regulatory-network analysis and retained WFDC2 as a secondary epithelial feature. Tissue Western blotting and immunohistochemistry were supplemented by H3K18cr detection and p65 immunoprecipitation to examine histone and p65-associated crotonylation signals.

We then used hdWGCNA, pseudotime, cell-communication analysis, *in silico* SDC4 perturbation, and transcriptome-derived metabolic scores to characterize associated epithelial programs. This study was designed to connect complementary observations across metabolic, transcriptional, and protein measurements, and to formulate a testable Kcr-p65-SDC4 association model rather than establish a regulatory cascade.

## Materials and methods

### Clinical data collection and statistical analysis

This retrospective study included 152 participants: 76 patients with adenomyosis (AM) and 76 controls (Con). Demographic variables, lipid profiles, routine blood indices, comorbidities, reproductive history, and recent medication use were extracted from medical records.

Baseline characteristics were analyzed in R using gtsummary and treated as descriptive, unadjusted comparisons. Continuous variables were summarized as mean ± SD or median [IQR] and compared with independent *t* tests or Mann-Whitney U tests, as appropriate; categorical variables were compared using chi-square or Fisher exact tests. Each clinical laboratory outcome was then modeled with AM status, age, parity (0, 1, 2, or ≥3), uterine fibroids, pelvic inflammatory disease, and any recent hormone use. Complete-case linear regression with HC3 robust standard errors yielded adjusted mean differences, 95% confidence intervals (CIs), and two-sided *P* values. Benjamini-Hochberg (BH) correction was applied across 17 laboratory outcomes and, separately, within the four-lipid family. Sensitivity analyses additionally adjusted for BMI, restricted the analysis to the overlapping age range of 27–48 years, modeled log-transformed lipid outcomes with a natural spline for age, and used propensity-score overlap weighting. All four lipid outcomes were reported irrespective of significance (Supplementary Tables S7a−7i).

### Public endometrial metabolomics reanalysis

The published OMIX007190 endometrial-tissue matrix and metadata (30) were reanalyzed for 56 AM patients and 97 healthy controls; endometriosis samples were excluded. Covariate-adjusted analysis used log2 abundance with adjustment for age, BMI, menstrual-cycle phase, parity, and hormone treatment. The reanalysis was used to identify lysine-related and lipid-related tissue metabolite patterns that could be compared with the new crotonyl-CoA and Kcr measurements. Feature-level results are provided in Supplementary Tables S9a, 9e.

### New tissue metabolite measurements

A targeted central-metabolism panel was measured in tissue from six AM patients and six controls, each sample representing a different patient. Differential analysis was performed on log2 concentrations using two-sided Welch tests. Acetyl-CoA and glutaryl-CoA were not quantifiable under the assay conditions. Processed metabolite-level differential-analysis results are provided in Supplementary Tables S10a, 10b.

### Crotonyl-CoA immunoassay

Tissue homogenates from seven AM patients and seven controls, all different patients within this assay, were examined using a research-use crotonyl-CoA ELISA kit (UpingBio, YPY23976). Three OD450 readings per sample were averaged before concentration estimation. Values were estimated against the supplied standard curve and reported on the assayed-homogenate concentration scale. Group comparisons used two-sided Welch tests at the patient level; processed assay summary values are provided in Supplementary Table S11.

### Data collection and preprocessing of scRNA-seq data

Raw single-cell count matrices were obtained from GEO datasets GSE218044 and GSE214411, including tissues from four AM patients and seven controls. Data processing was performed with Seurat v5. Cells with fewer than 800 detected genes or more than 15% mitochondrial transcripts were excluded, doublets were removed with DoubletFinder, and the remaining cells were log-normalized before highly variable genes were selected.

### Clustering and UMAP visualization

Harmony was used to correct batch effects across datasets. The integrated matrix was subjected to principal component analysis, and the first 10 principal components were used to construct the shared nearest-neighbor graph. Clusters were resolved by tuning the resolution parameter, yielding 22 transcriptomic clusters that were visualized by UMAP.

### Cell type annotation and marker gene analysis

Cell identities were assigned by comparing cluster-specific markers from Seurat FindMarkers with CellMarker, BioLegend, and published single-cell references. Cell-type proportions and disease-associated expression changes were compared between AM and Con groups. Feature plots, dot plots, and heatmaps were used to verify marker specificity and summarize the functional landscape of annotated cell populations.

### Kcr-related transcriptional score assessment

A 36-gene Kcr-related module was curated from GeneCards and published literature (Supplementary Table S1). A Kcr-related transcriptional score was estimated with five scoring methods: AddModuleScore, AUCell, UCell, singscore, and ssGSEA. Normalized scores were integrated into a composite score for cell-type and lineage-level comparisons. These expression-derived scores were not interpreted as direct measurements of protein crotonylation.

Cells were stratified by the composite Kcr-related transcriptional score into Low (< 25th percentile), Median, and High (>75th percentile) groups. These groups were projected onto UMAP coordinates to identify populations enriched for the gene-expression signature, with emphasis on epithelial and stromal compartments.

### Differential gene expression analysis based on Kcr-related transcriptional scores

Kcr-associated transcripts were identified by comparing High- and Low-Kcr cells with Seurat FindMarkers. The analysis prioritized genes upregulated in the High-Kcr group, including PAEP and SLC15A1.

### Network modeling via hdWGCNA

The hdWGCNA framework was applied to single-cell transcriptomes to identify modules associated with the Kcr-related transcriptional score. A soft-thresholding power of 8 was selected according to scale-free topology and connectivity metrics, and co-regulated genes were grouped into color-coded modules by hierarchical clustering of the topological overlap matrix. Module eigengenes and kME values were used to define module activity and intramodular hub genes.

### Functional enrichment and intersected gene characterization

Kcr-related candidate genes were defined as the overlap between high-Kcr upregulated genes and hdWGCNA module members. Venn diagrams and heatmaps were used to display the intersected genes and their expression differences between AM and Con groups. Gene-set variation analysis (GSVA) was additionally performed to characterize pathway-level differences between AM and control epithelial cells.

Functional enrichment of the intersected gene set was performed with clusterProfiler for Gene Ontology biological process, cellular component, molecular function, and KEGG pathway categories.

### Bulk data aggregation

After batch correction, expression matrices from GSE68870, GSE78851, GSE171653, GSE185392, GSE190580, GSE193928, and GSE244236 were merged, yielding 164 samples: 89 AM samples and 75 controls. Of the 106 single-cell-derived Kcr-score-associated candidates, 89 were represented in the bulk matrix. Samples were stratified by group and randomly divided at a 7:3 ratio into a training set of 114 samples (62 AM and 52 controls) and a held-out internal test set of 50 samples (27 AM and 23 controls) using seed 20260812.

### Multi-algorithmic feature selection architecture

LASSO, Random Forest, SVM-RFE, XGBoost, and Boruta were used for exploratory feature prioritization within the training set. LASSO and the final Random Forest, SVM, and Boruta-based Random Forest models used repeated five-fold cross-validation with three repeats; SVM-RFE used five-fold resampling to select feature-set size, and XGBoost used stratified five-fold cross-validation. SDC4 was selected in all five data-driven procedures; WFDC2 was selected by LASSO and SVM-RFE only. The held-out test set was used for a final internal performance assessment, and the models were interpreted as exploratory feature-prioritization tools (Supplementary Tables S4, S8b).

### Trajectory and cell communication analysis

CytoTRACE and Monocle were used to infer differentiation potential and pseudotime trajectories of SDC4+/- and WFDC2+/- epithelial cells. CellChat was then used to quantify ligand-receptor communication among epithelial subgroups and stromal, endothelial, and immune cells.

### Transcription factor prediction

Potential upstream transcription factors of SDC4 were predicted with NetworkAnalyst using TF-gene interaction resources and OmniPath-supported regulatory information. The resulting local network was ranked by topological metrics to prioritize candidate regulators.

### Western blotting

#### Protein extraction and quantification

Total protein was extracted from frozen AM and control tissues (*n* = 3 per group) using RIPA buffer with protease inhibitors. Lysates were cleared by centrifugation at 12,000 × g for 15 min at 4 °C, and protein concentrations were measured with a BCA assay.

#### SDS-PAGE and transfer

Protein samples (5 μL per lane) were separated by 10% SDS-PAGE and transferred to nitrocellulose membranes. Membranes were blocked for 20 min at room temperature before antibody incubation.

#### Antibody incubation

The membranes were incubated overnight at 4 °C with the following primary antibodies (all at 1:1000 dilution):

Rabbit anti-SDC4 (Cat# 11820-1-AP, Proteintech, China).

Rabbit anti-NF-κB p65 (Cat# 80979-1-RR, Proteintech, China).

Rabbit anti-GAPDH (Cat# db11729, Diagbio, China).

Mouse anti-Crotonylated Lysine (Cat# MA5-55992, Thermo Fisher Scientific, USA).

After washing three times with TBST, the membranes were incubated with HRP-conjugated goat anti-rabbit or anti-mouse secondary antibodies (1:20,000 dilution; Affinity Biosciences, China) for 1 h at room temperature.

#### Visualization and quantitative analysis

Bands were detected with enhanced chemiluminescence on a Tanon 5200 imaging system and quantified in ImageJ. Target protein levels were normalized to GAPDH.

### Histone and p65-associated crotonylation assays

H3K18cr and total histone H3 were assessed by Western blotting in three tissue specimens per group. H3K18cr/total H3 ratios were calculated from the selected bands, normalized to the control mean, and compared with a two-sided Welch *t* test.

For p65-associated Kcr, p65 was immunoprecipitated from tissue lysates and the recovered material was probed for Pan-Kcr and p65. IgG and input controls were included. The Pan-Kcr signal was normalized to recovered IP-p65 and expressed relative to control. Because there was one IP measurement per group, the IP comparison was reported descriptively. Densitometry values and uncropped image sources accompany Supplementary Figure S2 and the submitted source-data package.

### Immunohistochemistry (IHC)

#### Tissue preparation and antigen retrieval

FFPE sections (4 μm) from AM and control tissues (*n* = 5 per group) were deparaffinized, rehydrated, and subjected to EDTA antigen retrieval (pH 9.0, 20 min). Endogenous peroxidase activity was blocked with 3% H2O2 for 15 min.

#### Antibody incubation and staining

After blocking with 5% goat serum for 30 min, the sections were incubated overnight at 4 °C with primary antibodies against WFDC2, Pan-Kcr, SDC4, NF-κB p65, P-FAK, and P-Smad3.

Pan-Kcr (1:500; Thermo Fisher Scientific).

SDC4 (1:200; Proteintech).

NF-κB p65 (1:200; Proteintech).

P-FAK (Tyr397) (1:200; Cat# YM8684, ImmunoWay).

P-Smad3 (Ser423/425) (1:200; Cat# db13792, Diagbio).

WFDC2 (HE4) (1:200; Proteintech).

The sections were then washed and incubated with HRP-conjugated secondary antibodies for 1 h. Staining was visualized using a DAB Chromogen Kit, followed by counterstaining with hematoxylin.

### Computational gene perturbation analysis

SDC4-associated regulatory changes were predicted with scTenifoldKnk. AM epithelial cells and the top 5,000 highly variable genes plus SDC4 were used to construct gene regulatory networks. The observed and *in silico* SDC4-perturbed states were compared by manifold alignment, and genes predicted to be affected were identified using chi-square tests with Benjamini-Hochberg correction.

Genes identified as perturbed by the computational model were analyzed using GO, KEGG, and Reactome enrichment in clusterProfiler. Sankey plots were generated with ggsankey to link predicted genes with enriched terms. These analyses describe model predictions and do not substitute for an experimental SDC4 knockout.

The SDC4-centered ego network was visualized with plotKO using a weight quantile threshold of q = 0.997.

### Transcriptome-inferred metabolic pathway analysis

Metabolism-related pathway scores were derived with scMetabolism using the KEGG metabolism gene set and AUCell algorithm. Scores were calculated at whole-cell and epithelial-subcluster levels. Because these scores are based on pathway-gene expression, they do not directly measure metabolite abundance or metabolic flux.

Pathway-gene scores were compared between AM and control cells and across epithelial subgroups using the existing Seurat and rank-based analyses.

## Results

### Comparison of clinical baseline characteristics and metabolic indicators between AM and Con

The workflow is shown in Figure 1. The study included 76 AM patients and 76 controls (Table 1). AM patients were older than controls (median age, 48.00 vs. 33.00 years; *P* < 0.001) and had higher BMI (24.23 vs. 21.60; *P* < 0.001). Uterine volume and dysmenorrhea duration were also greater in the AM group (both *P* < 0.001).

**Figure 1:**
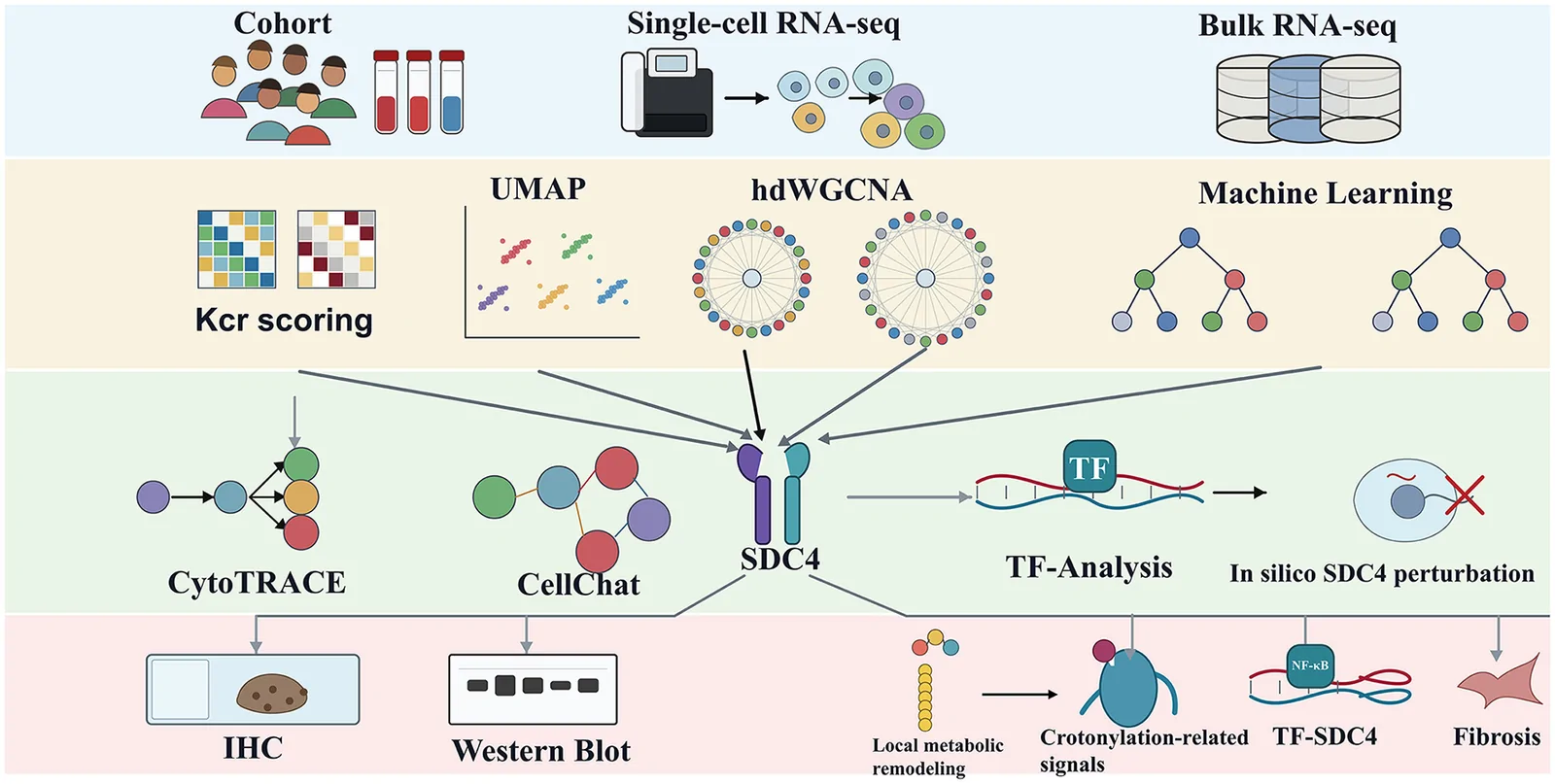
Study workflow. Clinical reanalysis and tissue metabolomic observations motivated assessment of crotonyl-CoA-associated assay signals and protein crotonylation. Single-cell and bulk-transcriptomic analyses prioritized epithelial features, followed by tissue protein assessments and computational investigation of associated programs. Arrows indicate the order of investigation, not experimentally demonstrated causal relationships.

**Table 1:** Unadjusted descriptive comparison of clinical baseline characteristics and laboratory indicators between patients with adenomyosis and controls.

Characteristics	AM *N* = 76	Con *N* = 76	Total (*N* = 152)^a^	*p*-value^b^
Age (year)	48.00 (45.00, 51.00)	33.00 (29.50, 38.00)	40.50 (33.00, 48.00)	< 0.001
BMI	24.23 (22.75, 26.67)	21.60 (19.53, 24.18)	23.44 (20.31, 25.67)	< 0.001
Uterine volume (cm^3^)	115.65 (81.36, 168.77)	55.33 (42.06, 68.91)	72.55 (51.49, 115.65)	< 0.001
Total cholesterol (TC)	4.10 (3.58, 4.72)	3.71 (3.45, 4.13)	3.85 (3.49, 4.39)	0.002
Triglycerides (TG)	1.22 (0.89, 1.47)	0.88 (0.62, 1.10)	1.01 (0.74, 1.33)	< 0.001
HDL-C	1.18 (1.02, 1.41)	1.22 (1.08, 1.42)	1.20 (1.05, 1.42)	0.230
LDL-C	2.58 (2.17, 2.95)	2.30 (2.01, 2.60)	2.43 (2.08, 2.80)	0.004
LDH	180.65 (± 36.57)	174.26 (± 35.00)	177.34 (± 35.78)	0.292
ALT	15.00 (11.00, 19.00)	11.00 (7.50, 17.00)	13.00 (9.00, 19.00)	0.001
AST	20.50 (17.00, 25.00)	18.50 (15.00, 24.00)	20.00 (16.00, 24.00)	0.043
Albumin	42.40 (39.60, 45.20)	43.80 (41.00, 46.00)	43.00 (40.20, 45.60)	0.071
Hemoglobin	112.00 (90.00, 128.50)	125.50 (114.50, 137.00)	121.00 (97.50, 134.50)	0.001
White blood cell count	5.32 (3.83, 6.65)	6.98 (5.18, 9.29)	5.77 (4.56, 8.29)	< 0.001
Menstrual cramps duration (year)	0.63 (0.00, 3.00)	0.00 (0.00, 0.00)	0.00 (0.00, 1.00)	< 0.001
Number of births				< 0.001
P0	1 (1.5%)	24 (33%)	25 (18%)	
P1	12 (18%)	20 (27%)	32 (23%)	
P2	33 (49%)	24 (33%)	57 (40%)	
P3+	22 (32%)	5 (6.8%)	27 (19%)	
Number of miscarriages				0.168
A1	22 (51%)	23 (68%)	45 (58%)	
A2+	21 (49%)	11 (32%)	32 (42%)	
Uterine fibroids	24 (32%)	1 (1.3%)	25 (16%)	< 0.001
Pelvic inflammatory disease	19 (25%)	5 (6.6%)	24 (16%)	0.003
Other gynecological history	37 (49%)	19 (25%)	56 (37%)	0.004
Renal insufficiency	2 (2.6%)	0 (0%)	2 (1.3%)	0.497
Systemic lupus erythematosus	2 (2.6%)	0 (0%)	2 (1.3%)	0.497
Hypertension	5 (6.6%)	0 (0%)	5 (3.3%)	0.058
Other immune system diseases	3 (3.9%)	1 (1.3%)	4 (2.6%)	0.620
Used GnRH-a recently (within 3 months)?	8 (11%)	0 (0%)	8 (5.3%)	0.006
Levonorgestrel intrauterine system use	7 (9.2%)	0 (0%)	7 (4.6%)	0.014
Taking oral contraceptives?	1 (1.3%)	0 (0%)	1 (0.7%)	>0.999
Take metformin?	1 (1.3%)	0 (0%)	1 (0.7%)	>0.999
Taking other hormone medications?	10 (13%)	1 (1.3%)	11 (7.2%)	0.009

^a^Median (Q1, Q3); Mean (± SD); *n* (%). ^b^Wilcoxon rank sum test; Welch Two Sample *t*-test; Wilcoxon rank sum exact test; Fisher's Exact Test for Count Data with simulated *p*-value.

Unadjusted comparisons showed higher total cholesterol, triglycerides, LDL-C, ALT, and AST and lower hemoglobin in AM. After adjustment for age, parity, uterine fibroids, pelvic inflammatory disease, and recent hormone use, the associations with total cholesterol, triglycerides, HDL-C, and LDL-C were not statistically supported. The adjusted ALT difference was −0.376 U/L (95% CI, −6.624 to 5.872; *P* = 0.905), AST was 0.337 U/L (95% CI, −3.994 to 4.668; *P* = 0.878), and hemoglobin was −10.86 g/L (95% CI, −23.30 to 1.59; *P* = 0.087; Table 2; Supplementary Tables S7a−7i). These clinical data redirected the analysis toward tissue-level metabolic remodeling.

**Table 2:** Covariate-adjusted clinical laboratory differences between AM and controls.

Outcome	*n*	Adjusted difference (95% CI)	*P* value	BH-FDR
Total cholesterol	141	0.089 mmol/L (−0.539 to 0.717)	0.780	0.933
Triglycerides	141	0.290 mmol/L (−0.103 to 0.682)	0.147	0.501
HDL-C	141	−0.023 mmol/L (−0.209 to 0.164)	0.809	0.933
LDL-C	141	0.018 mmol/L (−0.408 to 0.444)	0.933	0.933
ALT	141	−0.376 U/L (−6.624 to 5.872)	0.905	0.933
AST	141	0.337 U/L (−3.994 to 4.668)	0.878	0.933
Hemoglobin	141	−10.856 g/L (−23.302 to 1.591)	0.087	0.435

Adjusted difference is AM minus control. Models include age, parity, uterine fibroids, pelvic inflammatory disease, and recent hormone use, with HC3 robust standard errors. The principal complete-case cohort comprised 141 participants. BH-FDR was calculated across all 17 laboratory outcomes; complete models and sensitivity analyses are provided in Supplementary Tables S7a−7i.

### Public endometrial metabolomics supports local lipid and lysine-related remodeling

The OMIX007190 tissue dataset showed a broader pattern of metabolic differences between AM and healthy control endometrium (Figure 2A; Supplementary Figure S1). In the covariate-adjusted analysis, lysine, 5-aminopentanoic acid, and 5-hydroxylysine were higher in AM, with adjusted log2 differences of 0.439, 0.660, and 0.614 and BH-FDRs of 0.0160, 1.89e-7, and 1.98e-5, respectively. Carnitine, stearic acid, and palmitic acid were also higher (adjusted log2 differences, 0.957, 0.594, and 0.237; BH-FDRs, 0.00417, 3.91e-9, and 0.00799). Conversely, Sph (d18:2), hexadecenedioylcarnitine, 2-hydroxystearic acid, LPE (16:0), and PC(40:8) were lower. These bidirectional findings support altered tissue lipid composition and lysine-related metabolite pools, not a uniform increase in lipid synthesis (Table 3; Supplementary Tables S9a, 9e). Together with the higher lysine-related metabolites, these findings provided a tissue-level metabolic context for the crotonyl-CoA and Kcr measurements.

**Figure 2:**
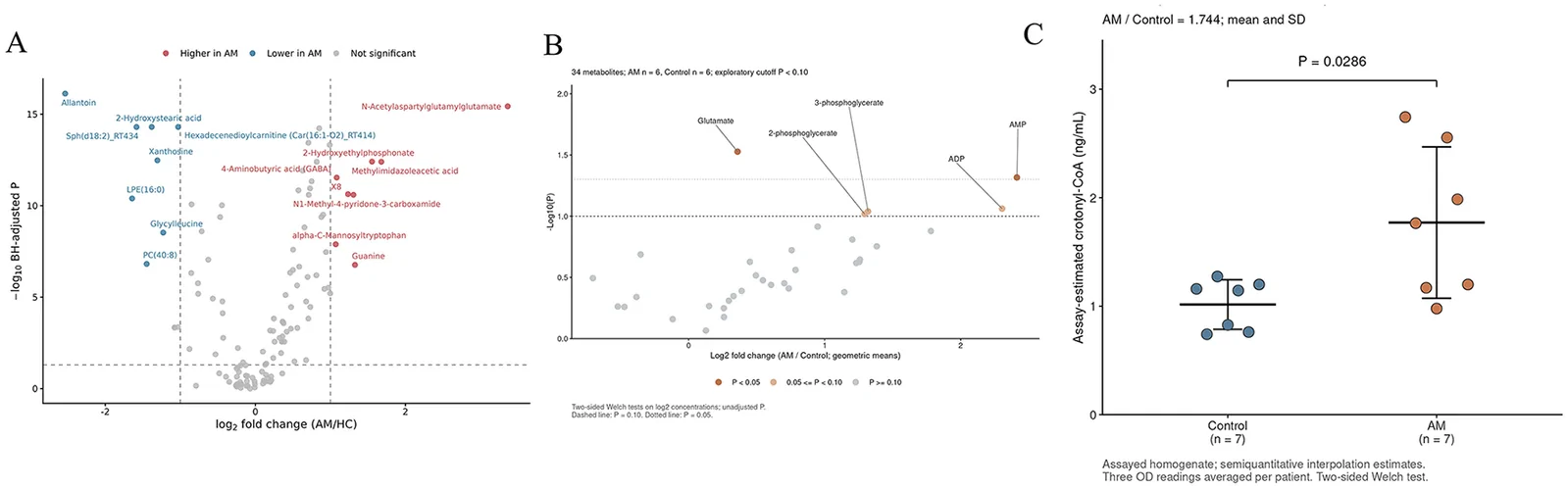
Tissue metabolite profiles and Crotonyl-CoA immunoassay estimates in adenomyosis. **(A)** Public OMIX007190 endometrial metabolomics (56 AM and 97 healthy controls), showing higher lysine-related metabolites together with bidirectional lipid-related changes. **(B)** New central-metabolism panel from six different patients per group, showing geometric mean ratios and two-sided Welch *P* values; glutamate, AMP, ADP, 3-phosphoglycerate, and 2-phosphoglycerate were higher in AM tissue. **(C)** Crotonyl-CoA immunoassay estimates from seven different patients per group. Dots represent patients, bars represent mean ± SD, and the two-sided Welch *P* value is 0.0286. Values are reported on the assayed-homogenate concentration scale.

**Table 3:** Selected covariate-adjusted endometrial metabolite differences in OMIX007190.

Metabolite feature	Adjusted log2 difference	*P* value	BH-FDR
Lysine	0.439	0.0097	0.016
5-Aminopentanoic acid	0.660	4.87e−08	1.89e−07
5-Hydroxylysine	0.614	6.54e−06	1.98e−05
Carnitine	0.957	0.00207	0.00417
Stearic acid	0.594	7.56e−10	3.91e−09
Palmitic acid	0.237	0.00432	0.00799
Sph (d18:2)_RT434	−1.316	2.23e−12	1.73e−11
Hexadecenedioylcarnitine [Car (16:1-O2)_RT414]	−1.023	3.7e−19	1.53e−17
2-hydroxystearic acid	−1.331	1.45e−18	4.48e−17
LPE(16:0)	−1.364	1.63e−11	1.12e−10
PC(40:8)	−3.931	2.98e−16	5.28e−15

Log2 abundance was modeled using limma with adjustment for age, BMI, menstrual-cycle phase, parity, and hormone treatment (56 AM and 97 healthy controls). Positive values indicate higher abundance in AM. The table includes increased and decreased lipid-related features and lysine-related metabolites. Complete feature results are supplied in Supplementary Tables S9a and S9e.

### New tissue measurements provide exploratory metabolic and crotonyl-CoA-associated evidence

In the newly measured central-metabolism panel, glutamate, AMP, ADP, 3-phosphoglycerate, and 2-phosphoglycerate showed higher geometric mean values in AM (geometric AM/control ratios, 1.283, 5.324, 4.939, 2.494, and 2.451; *P* = 0.0297, 0.0482, 0.0868, 0.0914, and 0.0959; Figure 2B; Supplementary Tables S10a, 10b). Acetyl-CoA and glutaryl-CoA were not quantifiable under the assay conditions, and crotonyl-CoA was measured separately by immunoassay.

The separate crotonyl-CoA immunoassay yielded higher estimates in AM than in controls (1.771 ± 0.698 vs. 1.015 ± 0.229 ng/ml of assayed homogenate; seven patients per group; mean ± SD). The mean difference was 0.756 ng/ml (95% CI, 0.104–1.407), with an AM/control mean ratio of 1.744 and two-sided Welch *P* = 0.0286 (Figure 2C). These findings indicate a higher crotonyl-CoA immunoassay signal in AM tissue and motivated investigation of tissue Kcr and its epithelial transcriptional associations.

### Single-cell transcriptomic landscape of AM and Con

Unsupervised clustering of scRNA-seq data identified six major cell populations by UMAP: stromal cells, vascular endothelial cells, T/NK cells, lymphatic endothelial cells, monocyte/macrophages, and epithelial cells (Figure 3A). Canonical markers supported these annotations, including VWF for vascular endothelial cells, EPCAM for epithelial cells, LUM for stromal cells, and CD3E for T/NK cells (Figure 3C).

**Figure 3:**
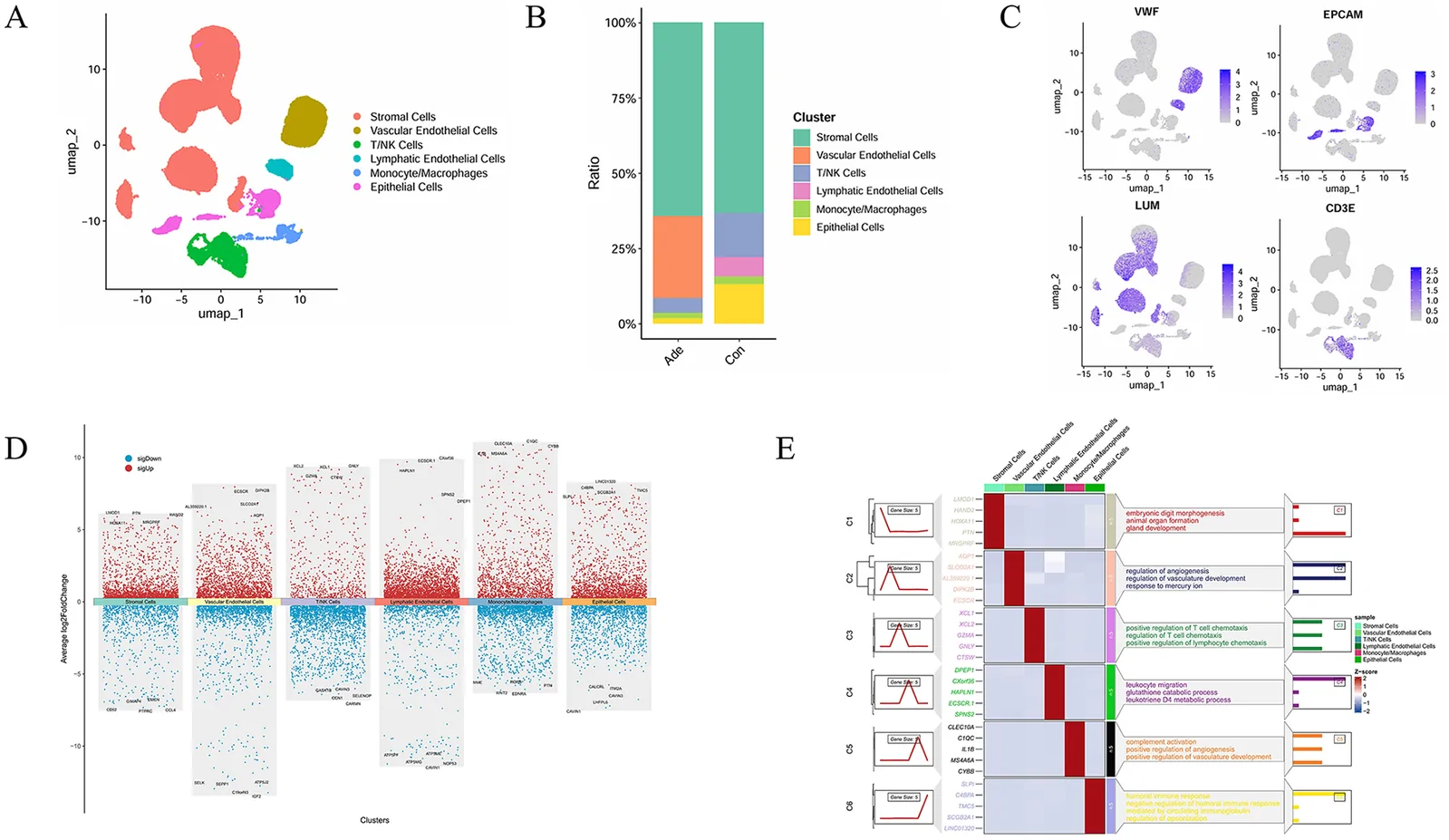
Single-cell transcriptomic landscape and functional characterization of adenomyosis and control tissues. **(A)** UMAP visualization identifying six major cell populations via unsupervised clustering: stromal cells, vascular endothelial cells, T/NK cells, lymphatic endothelial cells, monocyte/macrophages, and epithelial cells. **(B)** Cell proportion analysis comparing Ade and Con groups, summarizing observed cell-composition differences. **(C)** Feature plots of canonical marker genes validating the accuracy of cluster annotations, including VWF (vascular endothelial cells), EPCAM (epithelial cells), LUM (stromal cells), and CD3E (T/NK cells). **(D)** Overview of differential expression analysis identifying gene sets significantly up- or down-regulated across various cell types in the Ade group compared to the Con group. **(E)** Heatmap of top cluster-specific marker genes and corresponding GO functional enrichment analysis highlighting the biological characteristics of each population.

Cell-proportion analysis showed remodeling of the adenomyosis microenvironment, including increased vascular endothelial cells and altered epithelial and immune compartments in AM (Figure 3B). Differential expression analysis further indicated disease-associated transcriptional changes across multiple cell types (Figure 3D).

Functional enrichment of cluster-specific markers linked stromal cells to extracellular matrix organization, endothelial cells to angiogenesis, immune cells to activation and migration programs, and epithelial cells to differentiation, adhesion, and structural integrity (Figure 3E).

### Characterization of the single-cell Kcr-related transcriptional landscape in AM

To map Kcr-related transcriptional patterns at single-cell resolution, we curated 36 Kcr-related genes and scored cells using five complementary algorithms. Both individual and composite expression-derived scores showed marked heterogeneity across cell populations (Figures 4A, B).

**Figure 4:**
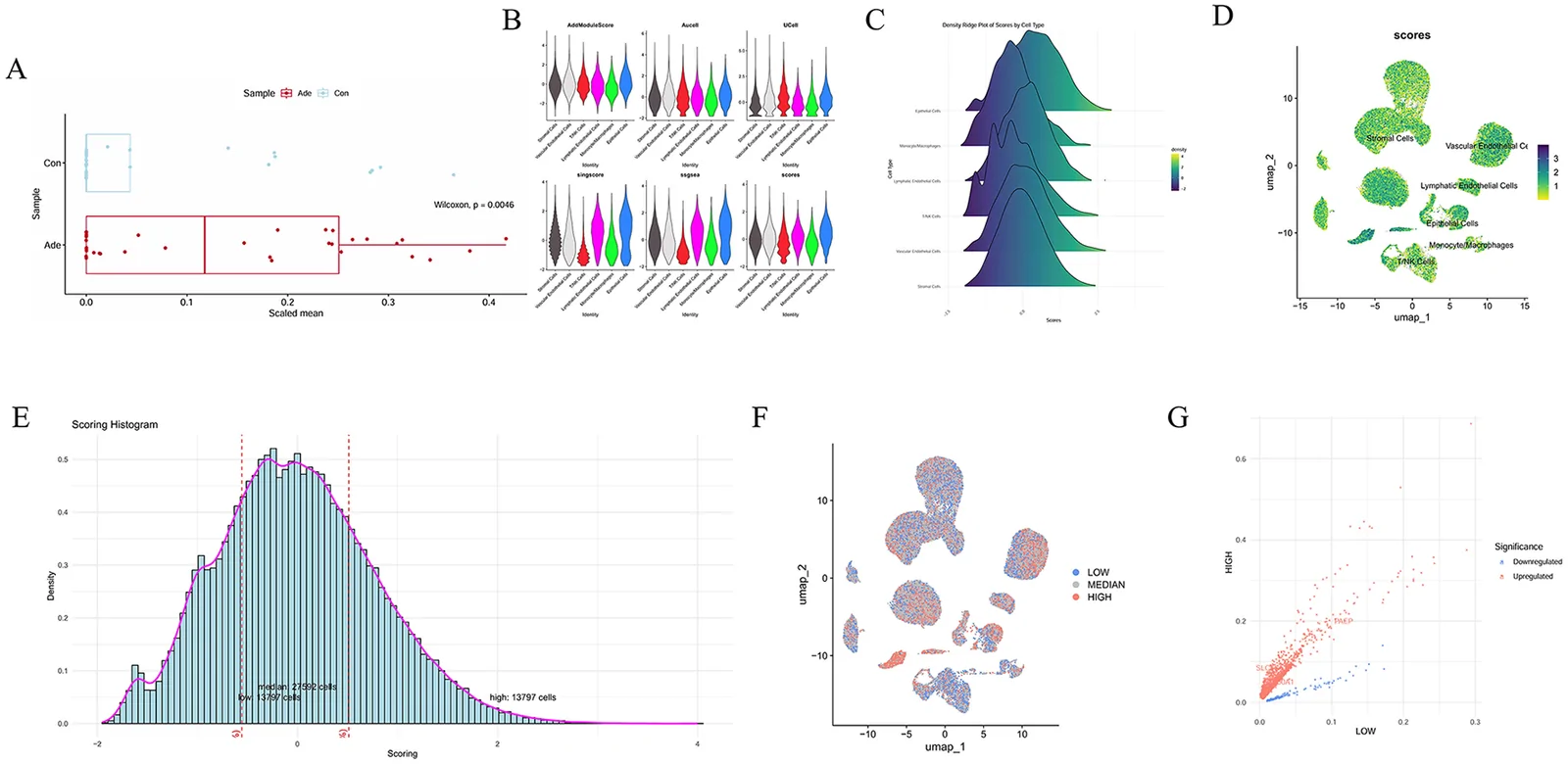
Single-cell characterization of Kcr-related transcriptional scores in adenomyosis. **(A)** Comparison of 36 Kcr-related genes between adenomyosis (Ade) and control (Con) groups. **(B)** Violin plots showing expression-derived Kcr-related scores across cell populations using AddModuleScore, AUCell, UCell, singscore, and ssGSEA. **(C)** Ridge plot of the integrated score. **(D)** Integrated score mapped onto UMAP space. **(E)** Classification into High, Median, and Low score groups by quartiles. **(F)** Score groups mapped onto UMAP. **(G)** Differential transcripts associated with the Kcr-related score. These scores reflect gene expression and are not direct measurements of protein crotonylation.

The composite Kcr-related transcriptional score was highest in epithelial cells, as shown by ridge plots and UMAP projection (Figures 4C, D). Quartile-based stratification classified cells into High-, Median-, and Low-score groups, with 13,797 cells each in the High- and Low-score groups and 27,592 cells in the Median-score group (Figure 4E).

When projected onto UMAP space, High-score cells were enriched among epithelial cells, whereas T/NK cells were mainly in the Low-score group. Stromal cells showed broader within-cluster heterogeneity. Differential expression between the High- and Low-score groups identified score-associated genes, including PAEP and SLC15A1 (Figures 4F, G; Supplementary Table S2).

### Construction of single-cell coexpression networks

hdWGCNA was used to characterize regulatory modules in AM tissues. A soft power threshold of 8 produced a scale-free network, and dynamic tree cutting identified 11 co-expression modules (Figures 5A, B). kME-based connectivity analysis nominated hub genes within each module (Figure 5C; Supplementary Table S3).

**Figure 5:**
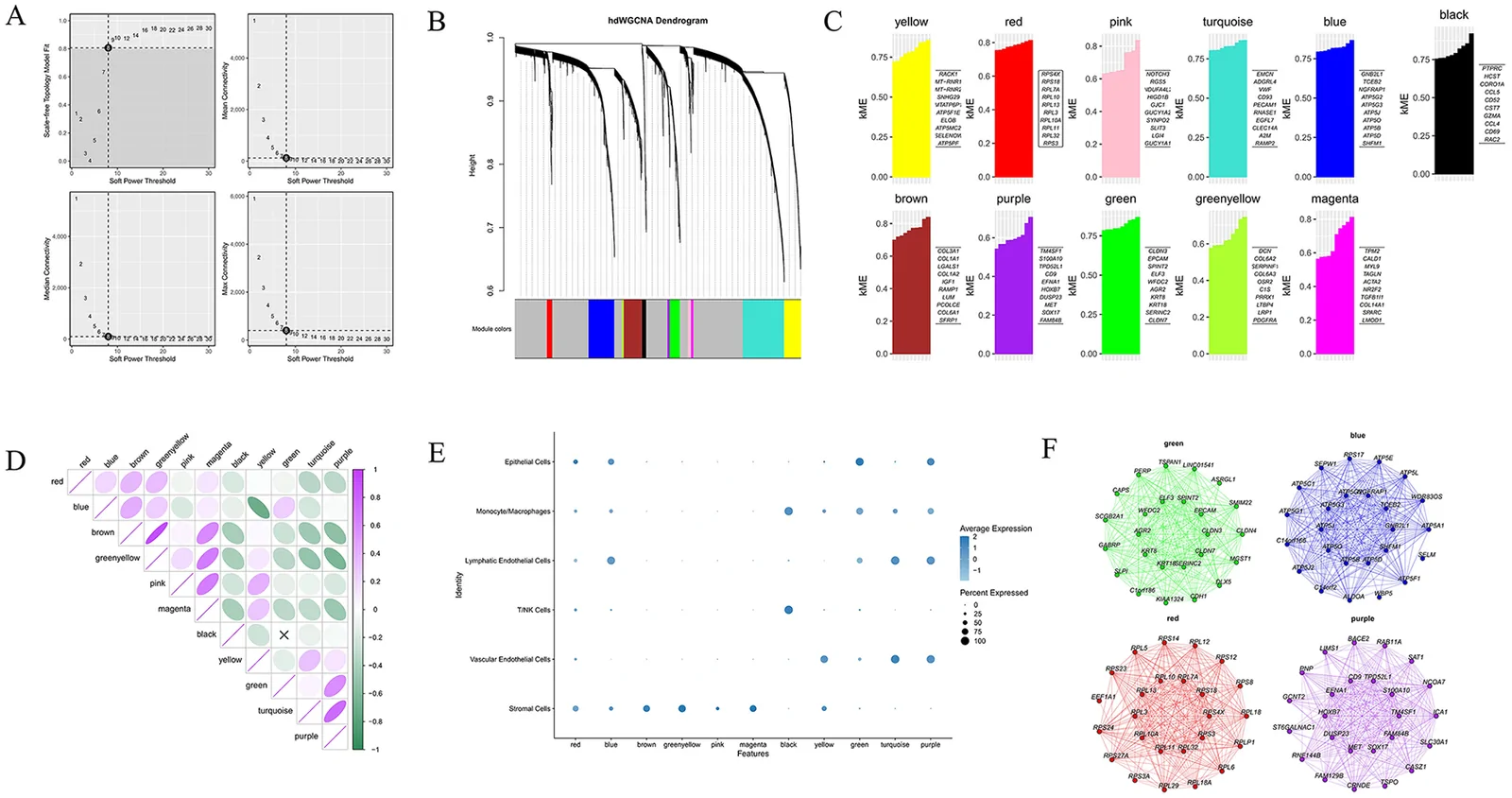
Construction of single-cell co-expression networks and identification of cell-type-specific modules via hdWGCNA. **(A)** Scale-free topology fitting index and mean connectivity analysis determining a soft-thresholding power of 8. **(B)** Cluster dendrogram identifying 11 distinct co-expression modules based on the dynamic tree-cut algorithm. **(C)** Module membership (kME) connectivity analysis identifying intramodular hub genes. **(D)** Correlation heatmap illustrating the relationships between various co-expression gene modules. **(E)** Dot plot mapping module eigengenes to cell types. **(F)** Network visualization of modules (green, blue, red, and purple) representing co-expression hubs associated with specific cell types in adenomyosis.

Mapping module eigengenes to cell populations showed that green and purple modules were enriched in epithelial cells, the brown module in stromal cells, and the blue module in lymphatic endothelial cells (Figures 5D, E). Network visualization then highlighted hub genes in epithelial- and stromal-related modules (Figure 5F).

### Identification and functional enrichment of Kcr-score-associated feature genes

Intersecting genes upregulated in the High-score group with hdWGCNA module members (kME > 0.4) yielded 106 Kcr-score-associated features (Figures 6A, B). GSVA further indicated pathway-level transcriptional differences between AM and control epithelial cells (Figure 6C).

**Figure 6:**
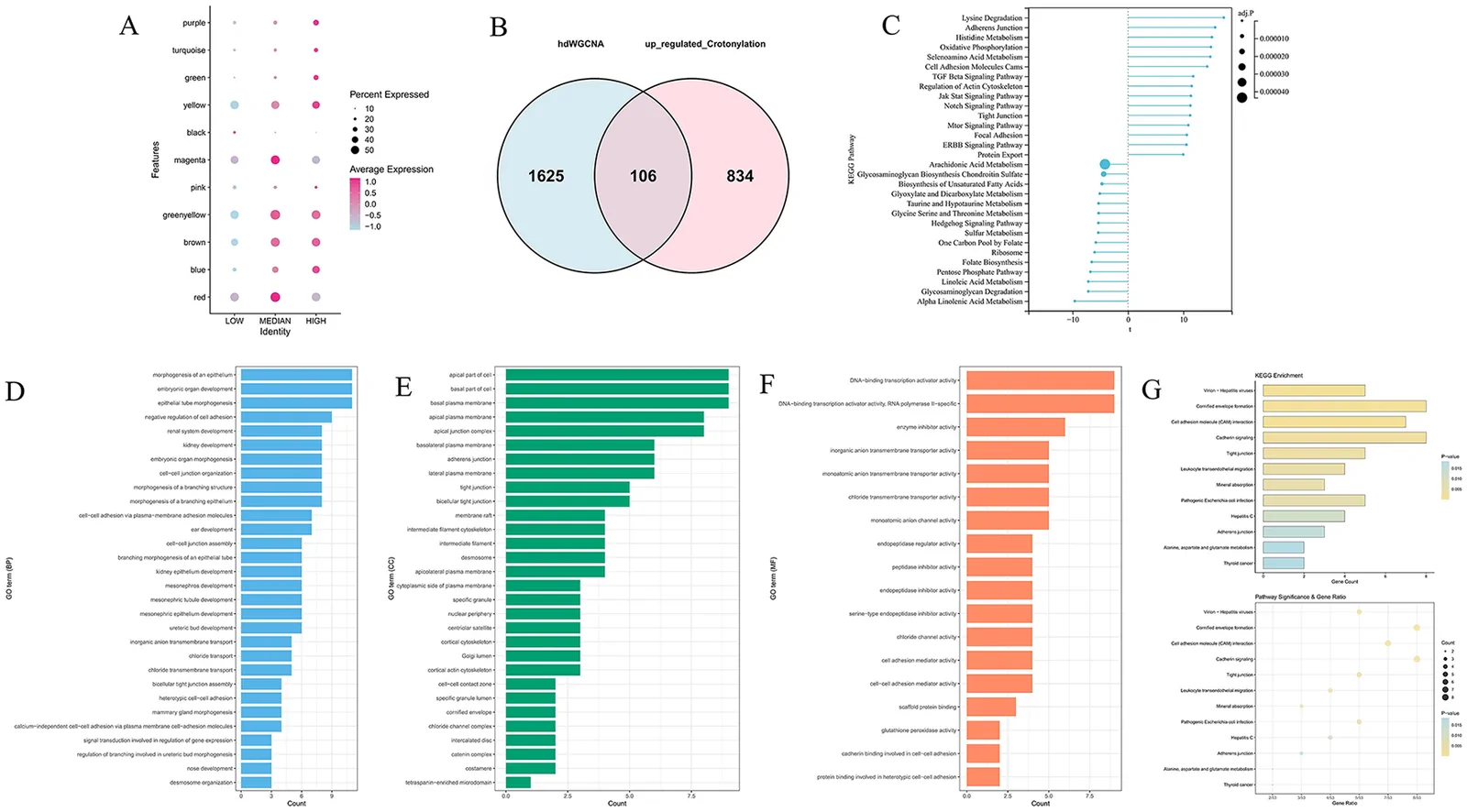
Identification and functional characterization of Kcr-score-associated epithelial features in adenomyosis. **(A)** hdWGCNA modules associated with higher Kcr-related transcriptional scores. **(B)** Intersection of transcripts higher in the High-score group with module members (kME > 0.4), yielding 106 score-associated features. **(C)** GSVA comparison between Ade and Con epithelial cells. **(D–F)** GO enrichment and **(G)** KEGG enrichment of the 106 features. These analyses describe transcriptional associations and do not measure protein crotonylation.

GO enrichment of the 106 feature genes supported epithelial remodeling, with biological processes related to epithelial morphogenesis and junction organization; cellular components including apical and basal cell regions and the apical junction complex; and molecular functions including transcriptional activator activity, cell-cell adhesion mediator activity, and cadherin binding (Figures 6D–F).

Enrichment of the 106 features included cadherin- and junction-related terms (Figure 6G), consistent with an association between the Kcr-related transcriptional signature and epithelial structural programs.

### Exploratory prioritization of AM features using multiple machine-learning algorithms

Of the 106 single-cell-derived candidates, 89 mapped to the bulk matrix and entered the revised training-set analysis. After tuning, the final LASSO, Random Forest, SVM-RFE, XGBoost, and Boruta feature sets contained 2, 20, 40, 18, and 16 genes, respectively (Figures 7A–G).

**Figure 7:**
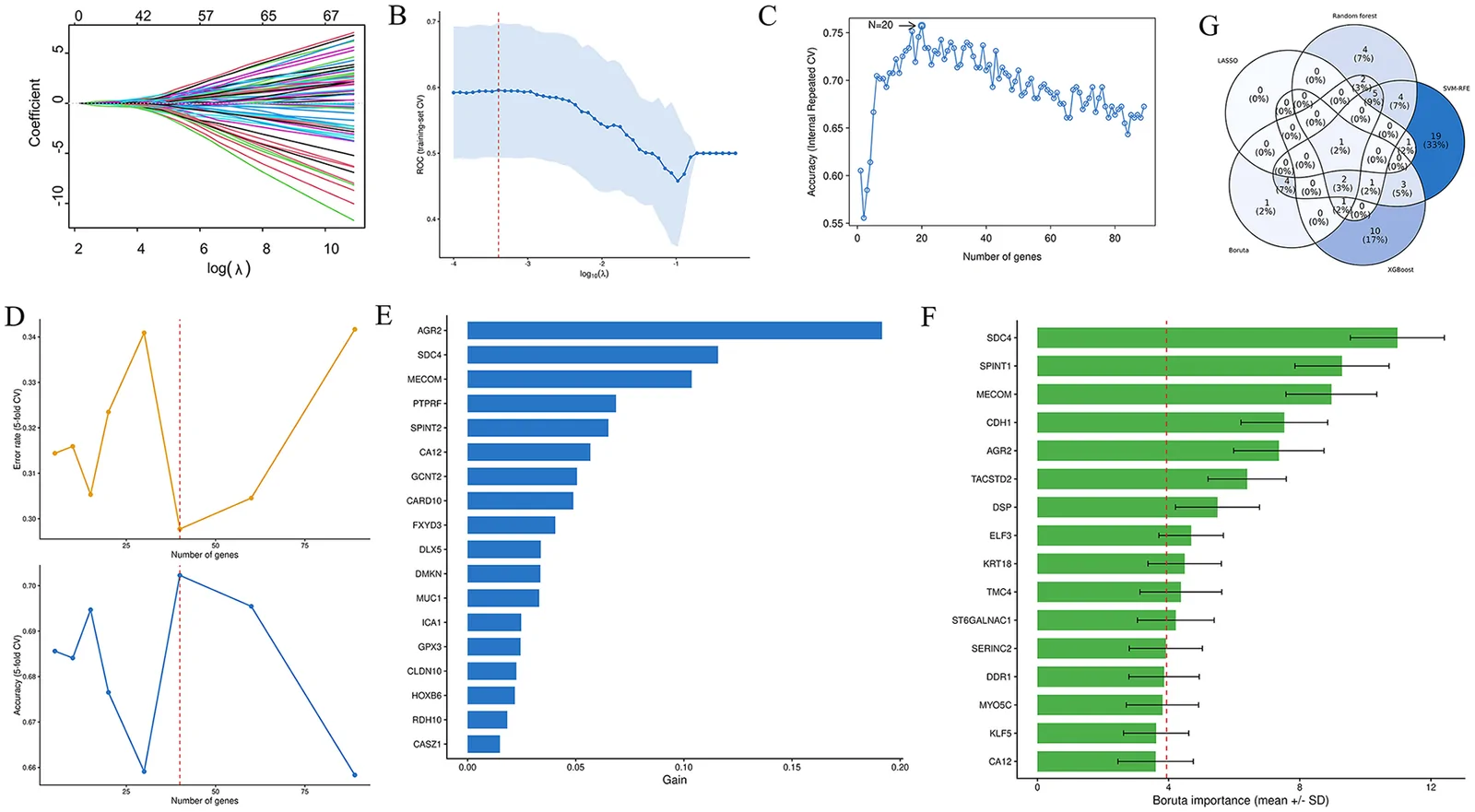
Five machine-learning screening procedures and data-driven feature overlap after a 7:3 training/test split. **(A)** LASSO coefficient path, **(B)** LASSO cross-validation, **(C)** Random Forest RFE accuracy curve, **(D)** SVM-RFE error and accuracy profiles, **(E)** XGBoost feature importance, **(F)** Boruta-based Random Forest feature importance, and **(G)** the data-driven Venn diagram. SDC4 was selected by all five data-driven procedures. WFDC2 was selected by the data-driven screens of LASSO and SVM-RFE only. These analyses were used for exploratory candidate prioritization.

SDC4 was selected by the data-driven screens of all five methods. WFDC2 was selected by the data-driven screens of LASSO and SVM-RFE only. These results support SDC4 as the primary exploratory candidate.

### Bulk-expression comparison and single-cell localization of prioritized features

In the merged bulk-transcriptomic cohort, SDC4 (*P* = 0.005) and WFDC2 (*P* = 0.02) were more highly expressed in AM than in control samples (Figure 8A).

**Figure 8:**
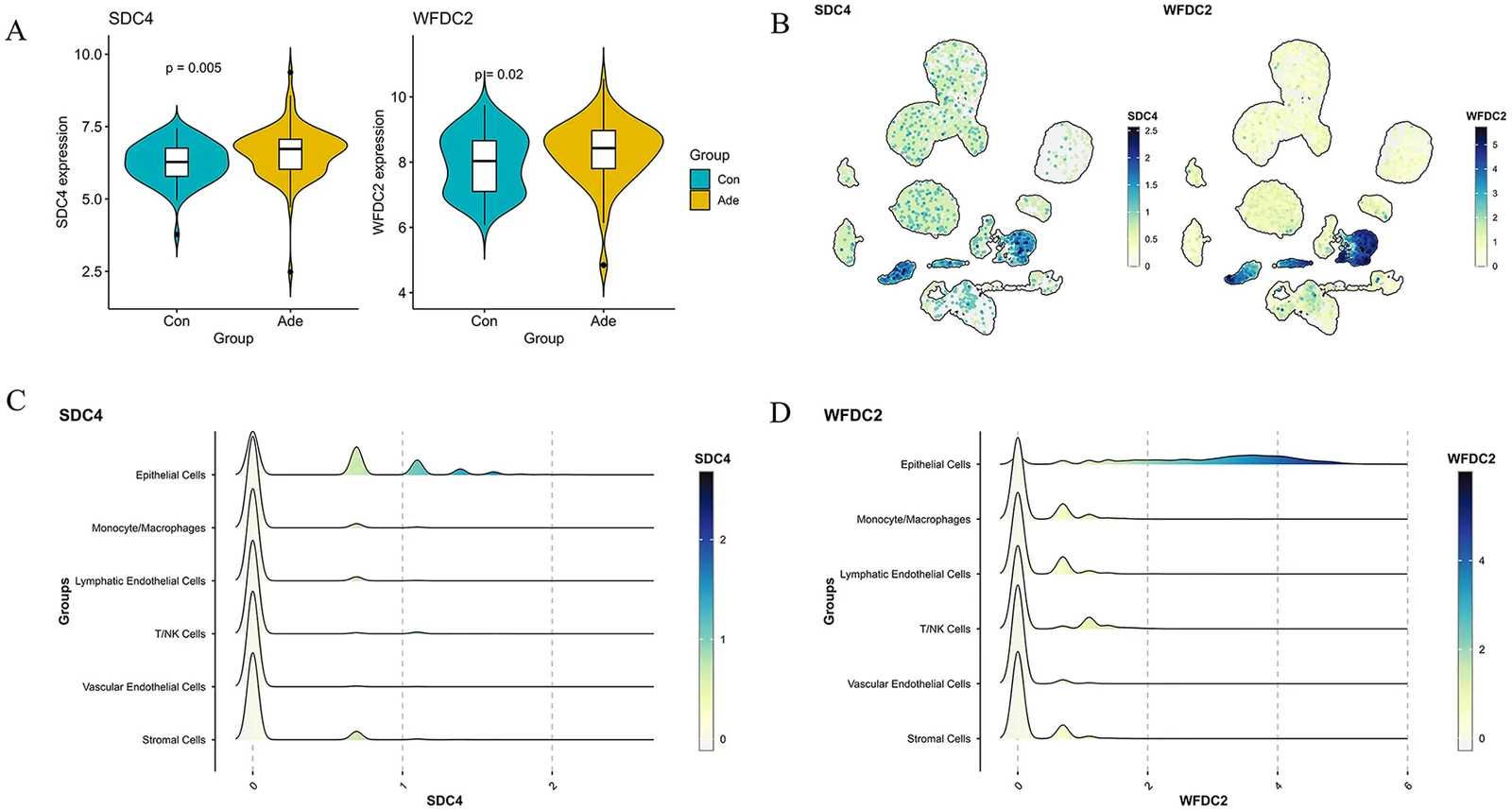
Bulk-expression comparison and single-cell distribution of prioritized features SDC4 and WFDC2. **(A)** Expression comparison in the merged bulk-transcriptomic cohort. **(B–D)** Single-cell localization showing epithelial enrichment of SDC4 and WFDC2.

Single-cell mapping localized SDC4 and WFDC2 predominantly to epithelial clusters (Figure 8B). SDC4 was low or undetectable in most immune and endothelial cells but abundant in epithelial cells and moderately expressed in stromal cells (Figure 8C). WFDC2 was broadly detectable but showed strongest enrichment in epithelial cells (Figure 8D).

### Inferred differentiation trajectories and CellChat patterns of SDC4+ and SDC4- epithelial cells

CytoTRACE assigned higher scores to SDC4+ epithelial cells, consistent with a less differentiated transcriptional state than that of SDC4- cells (Figures 9A, C). Monocle placed many SDC4+ cells near the early portion of the inferred trajectory from Cluster 1 toward Clusters 2 and 3 (Figures 9B, D). These algorithms order cells by transcriptional similarity and do not establish lineage ancestry.

**Figure 9:**
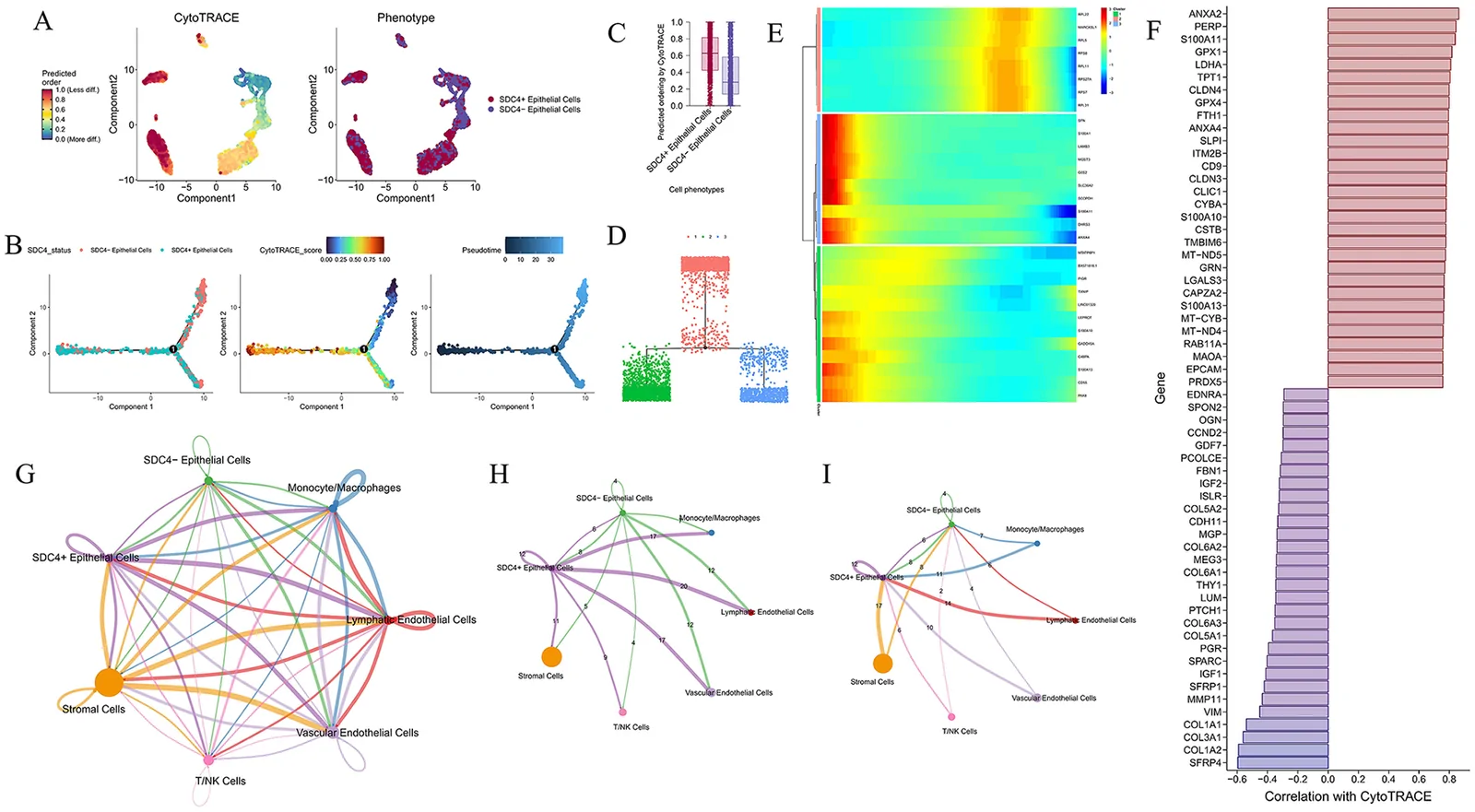
Differentiation trajectory and intercellular communication analysis of SDC4+ epithelial cells and SDC4− epithelial cells. **(A, B**) CytoTRACE-inferred differentiation scores and cell mapping of SDC4+ and SDC4− epithelial cells, and Monocle pseudotime trajectories colored by SDC4 status, CytoTRACE score, and pseudotime, respectively. **(C, D)** Boxplot comparison of CytoTRACE scores between SDC4+ and SDC4− epithelial cells, and distribution of epithelial clusters along the Monocle-inferred differentiation trajectory from Cluster 1 to Clusters 2 and 3, respectively. **(E)** Gene expression heatmap illustrating transcriptomic dynamics along the pseudotime axis. **(F)** Visualization of the top 30 genes showing positive and negative correlations with CytoTRACE differentiation scores. **(G)** Global intercellular communication network between epithelial subclusters and stromal, endothelial, and immune cell populations. **(H, I)** Quantitative split analysis showing the intensities of outgoing and incoming signaling for SDC4+ and SDC4− epithelial subclusters.

The pseudotime heatmap showed coordinated changes in gene modules along the inferred trajectory (Figure 9E). Correlation analysis identified the 30 genes most positively and negatively associated with CytoTRACE scores (Figure 9F).

CellChat predicted broader incoming and outgoing ligand-receptor interaction patterns for SDC4+ than for SDC4- epithelial cells (Figures 9G–I).

To compare predicted intercellular communication in the AM lesion microenvironment, CellChat was used to infer ligand-receptor networks linking SDC4+ and SDC4- epithelial cells with stromal, endothelial, and immune populations.

Aggregate scores were modestly higher for outgoing interactions, with a more pronounced difference in incoming interactions in SDC4+ epithelial cells (Figure 10G). Pathway-level differences included LIFR, CALCR, COMPLEMENT, GDF, SPP1, and GALECTIN among outgoing patterns and BMP, PERIOSTIN, EGF, GRN, PARs, and CypA among incoming patterns (Figure 10A). TWEAK, IGF, and MK also showed higher inferred incoming scores in SDC4+ than in SDC4- cells. These values are algorithm-derived communication probabilities rather than measurements of pathway activation.

**Figure 10:**
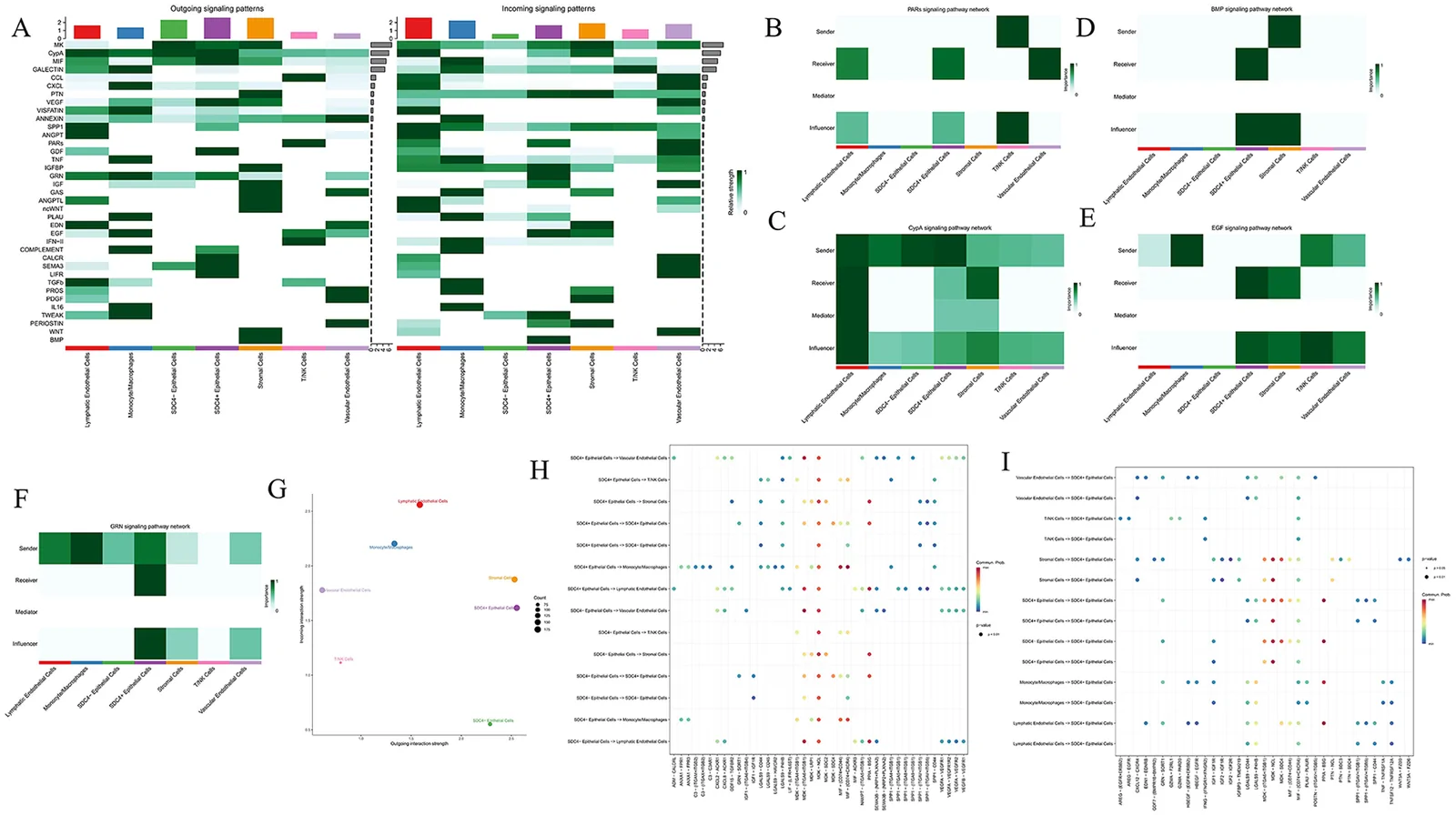
CellChat-based inference of signaling patterns and ligand-receptor interactions for SDC4 epithelial subclusters. **(A)** Heatmaps of outgoing and incoming signaling patterns across various cell types. **(B–F)** Heatmaps displaying network centrality measures including sender, receiver, mediator, and influencer roles for cell populations within specific signaling pathways: PARs **(B)**, CypA **(C)**, BMP **(D)**, EGF **(E)**, and GRN **(F)**. **(G)** Scatter plot summarizing the total outgoing and incoming interaction strengths of all identified cell types, including the SDC4+ and SDC4- epithelial subclusters. **(H)** Dot plot of specific ligand-receptor pairs illustrating outgoing signaling interactions from SDC4+ and SDC4- epithelial cells to other microenvironmental components. **(I)** Dot plot of specific ligand-receptor pairs depicting incoming signals received by SDC4+ and SDC4- epithelial cells from other cell populations.

CellChat network-role scores assigned T/NK cells as candidate senders in the PARs pathway, with SDC4+ epithelial and endothelial cells among the candidate receivers (Figure 10B). Predicted receiver or mediator roles in CypA included lymphatic endothelial, SDC4+ epithelial, and stromal cells (Figure 10C). Stromal cells were the predicted sender in BMP, whereas immune or endothelial populations contributed candidate EGF signals; SDC4+ epithelial cells had the highest predicted receiver centrality in GRN (Figures 10D–F).

Dot-plot analysis nominated candidate outgoing interactions from SDC4+ epithelial cells to monocytes/macrophages through C3-related, GDF15-TGFBR2, LGALS9-related, and SPP1-related pairs (Figure 10H). Candidate interactions with lymphatic endothelial cells included CXCL2/8-ACKR1, LIF-related, SPP1-integrin, and LGALS9-related pairs.

Candidate incoming interactions for SDC4+ epithelial cells included EDN1, GRN, HBEGF, MDK, and POSTN from endothelial cells; AREG and GZMA from T/NK cells; and GDF7, PTN, WNT5A, MIF, and IGF2 from stromal cells (Figure 10I). PPIA-BSG and SPP1-integrin pairs were also predicted from macrophage and lymphatic-endothelial populations. SDC4- epithelial cells showed a narrower predicted pattern that included MDK-ITGA6/ITGB1.

### Inferred differentiation trajectories and CellChat patterns of WFDC2+ and WFDC2- epithelial cells

CytoTRACE and Monocle were used to compare inferred differentiation states of WFDC2+ and WFDC2- epithelial cells. WFDC2- cells occupied both high- and low-CytoTRACE-score regions, whereas WFDC2+ cells were concentrated mainly in the middle and later portions of the inferred trajectory (Supplementary Figures S3A–D). This distribution indicates transcriptional heterogeneity but does not assign the subgroups to fixed stages of AM progression.

The pseudotime heatmap showed three expression modules along the inferred trajectory (Supplementary Figure S3E). ADGRF1, GPX3, ANXA4, PAX8, SPP1, CXCL14, and S100P were higher near the early pseudotime region, whereas RPS/RPL-family genes, COL3A1, SFRP4, HSPA1A, and SPDEF increased toward the later region. These changes describe pseudotime-associated transcription and were not interpreted as experimentally verified differentiation.

CellChat estimated higher overall communication strength for WFDC2+ than for WFDC2- epithelial cells (Supplementary Figures S3F, G), although the predicted outgoing pattern toward lymphatic endothelial cells was higher for WFDC2- cells and was similar between subgroups toward stromal cells. For inferred incoming interactions, T/NK and vascular endothelial cells contributed higher scores toward WFDC2- cells, whereas monocytes/macrophages and stromal cells contributed higher scores toward WFDC2+ cells; lymphatic-endothelial scores were similar (Supplementary Figure S3H). These are descriptive differences in predicted communication, not evidence of regulatory preference or disease-stage progression.

CellChat was then used to compare predicted pathway patterns and ligand-receptor interactions between WFDC2+ and WFDC2- epithelial states.

Pathway-level CellChat scores differed between WFDC2+ and WFDC2- subgroups (Supplementary Figure S4A). WFDC2- cells showed higher inferred outgoing patterns for CALCR, LIFR, IGF, GDF, SPP1, and VEGF, whereas WFDC2+ cells showed higher scores for COMPLEMENT, SEMA3, GALECTIN, MK, and CypA. For incoming patterns, WFDC2- cells had higher inferred scores for EDN, PARs, PLAU, SPP1, and CCL.

CellChat topology assigned WFDC2+ epithelial cells a candidate sender role in SEMA3 and WFDC2- cells a candidate sender role in LIFR and CALCR (Supplementary Figures S4B, C, E). Monocytes/macrophages and WFDC2+ epithelial cells contributed predicted outgoing COMPLEMENT signals (Supplementary Figure S4D). Stromal-to-WFDC2+ BMP and T/NK-to-WFDC2- PARs interactions were also inferred (Supplementary Figures S4F, G).

Ligand-receptor analysis nominated ADM-CALCRL, LIF-LIFR/IL6ST, SPP1-integrin, and IGF1-IGF1R pairs in the WFDC2- communication pattern (Supplementary Figure S4H). Candidate WFDC2+ interactions included SEMA3-NRP/PLXN, C3-integrin/C3AR1, LGALS9-related, and MDK-LRP1 pairs.

Predicted incoming interactions differed between WFDC2-defined subgroups, including EDN1-, GZMA-, and PTN-related pairs in WFDC2-negative cells and GDF7-, MDK-, and PPIA-related pairs in WFDC2-positive cells (Supplementary Figure S4I).

### Candidate transcription-factor analysis and tissue-level protein assessment

Transcription-factor network analysis nominated RELA/p65 and other NF-κB-family members as candidate regulators of SDC4 (Figure 11A). The original Western blots and representative immunohistochemical images showed tissue patterns involving SDC4, p65, Pan-Kcr, WFDC2, P-FAK, and P-Smad3 (Figures 11B–D). These are protein-abundance or staining observations, not measurements of promoter occupancy or regulatory order. In the additional histone experiment, H3K18cr normalized to total H3 was 1.94-fold higher in AM than in controls (three specimens per group; two-sided Welch *P* = 0.0161; Figures 11E, F; Supplementary Figure S2).

**Figure 11:**
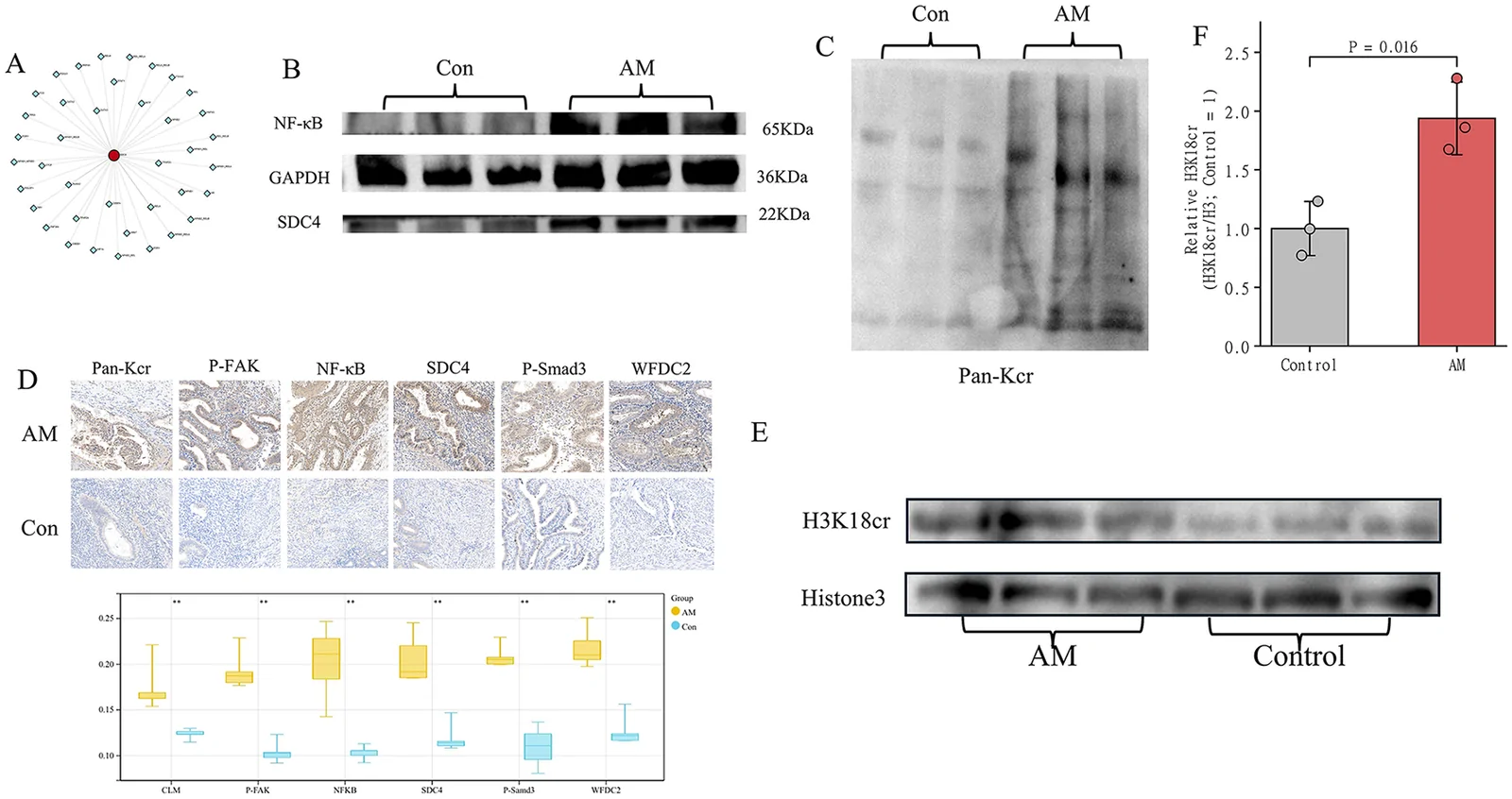
Candidate transcriptional regulators and tissue protein observations. **(A)** NetworkAnalyst nomination of SDC4 regulators. **(B, C)** Tissue Western blots for SDC4, p65, and Pan-Kcr, with GAPDH as the loading control (three specimens per group). **(D)** Representative IHC images for SDC4, p65, Pan-Kcr, WFDC2, P-FAK, and P-Smad3 in AM and control tissues (five patients per group). Panels B-D are descriptive and are shown without patient-level significance annotations. **(E)** H3K18cr and total H3 blots in the additional experiment. **(F)** H3K18cr/total H3 ratios normalized to the control mean (three specimens per group). Points show specimen-level values and bars show mean ± SD; two-sided Welch *t* test, nominal *P* = 0.0161. The AM/control mean ratio was 1.94. The p65 immunoprecipitation/Pan-Kcr comparison is reported in Supplementary Figure S2 and comprised one IP measurement per group; it should therefore be regarded as a preliminary descriptive observation only. Source values and the submitted uncropped H3 and p65-immunoprecipitation blots are provided in Supplementary Figure S2 and the accompanying source-data package.

In the p65 experiment, Pan-Kcr signal in the p65 immunoprecipitate, normalized to IP-p65, was 3.086-fold higher in the AM specimen than in the control specimen (Supplementary Figure S2). Because only one IP measurement was available per group, this finding should be regarded as a preliminary descriptive observation, and no patient-level or group-level inferential conclusion can be drawn. The observation is consistent with the presence of a p65-associated Kcr signal in AM tissue and warrants validation in additional independent specimens, together with site-specific and functional studies.

### Predicted SDC4-associated transcriptional changes identified by scTenifoldKnk

To explore SDC4-associated transcriptional programs, we used scRNA-seq data to simulate an *in silico* SDC4 perturbation and assessed the predicted changes in the epithelial regulatory network (Supplementary Table S5).

*In silico* SDC4 perturbation identified genes predicted to differ in network state, including ATOH8, ADGRF1, RXFP1, and GABRP (Figures 12A–C). The SDC4-centered network summarizes predicted relationships within epithelial transcriptional data (Figure 12D). The large modeled perturbation of SDC4 reflects the design of the simulation and should not be interpreted as independent validation of SDC4 function.

**Figure 12:**
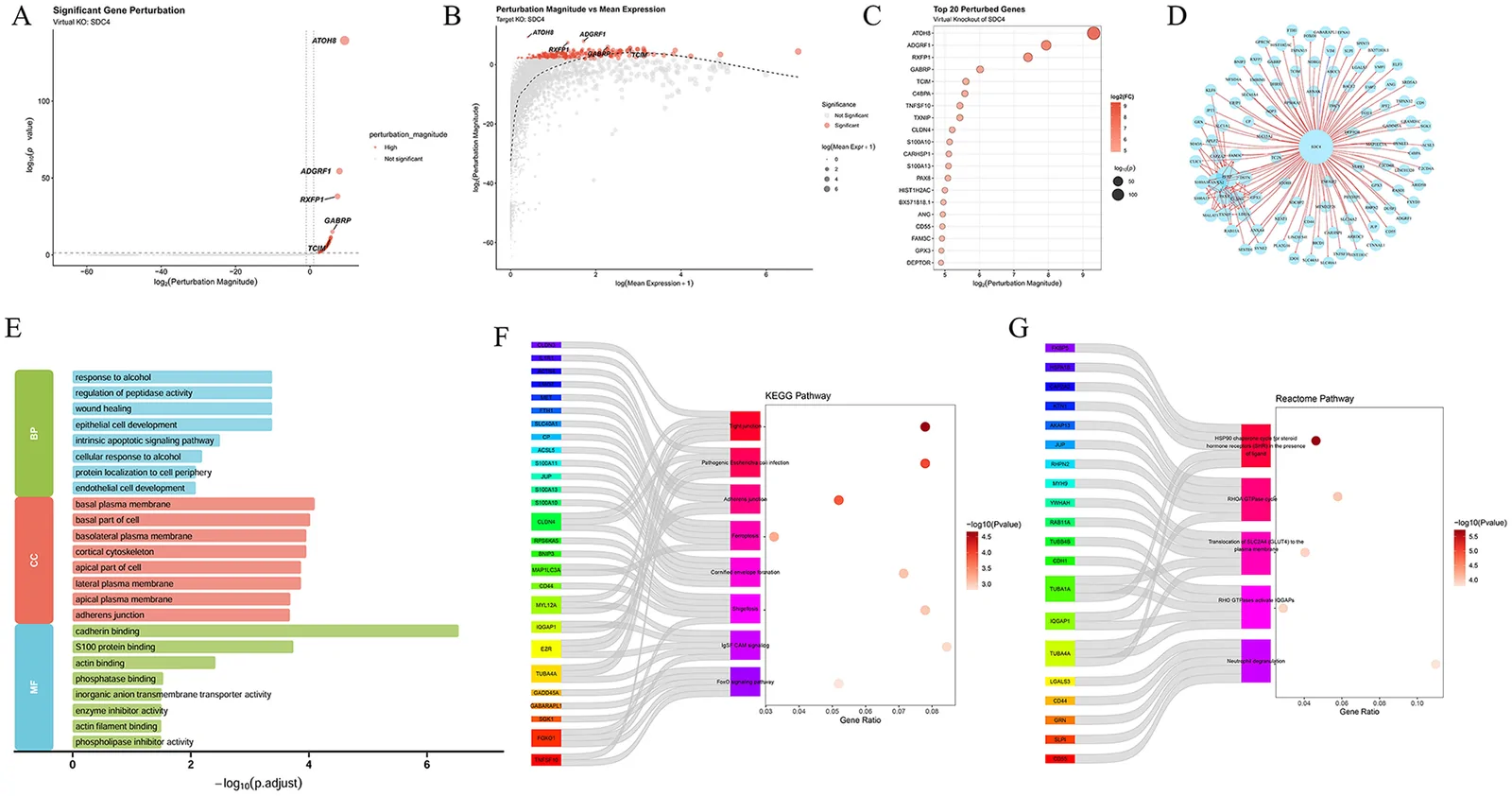
*In silico* SDC4 perturbation in epithelial cells using scTenifoldKnk. **(A, B)** Predicted network perturbations. **(C)** Top predicted perturbed genes, including ATOH8, ADGRF1, RXFP1, and GABRP. **(D)** SDC4-centered predicted network. **(E)** GO enrichment of the predicted gene set, grouped into biological processes, cellular components, and molecular functions. **(F, G)** KEGG and Reactome enrichment, including junction, RHO-GTPase, SLC2A4/GLUT4-translocation, and neutrophil-degranulation terms. These are network predictions, not observed fold changes following an experimental knockout.

GO enrichment of predicted perturbed genes included epithelial development and wound-healing terms in biological processes, basal plasma membrane and adherens junction terms in cellular components, and cadherin, S100-protein, and actin binding in molecular functions (Figure 12E).

Functional enrichment after computational SDC4 perturbation linked the predicted gene set to epithelial adhesion and junction pathways, including tight and adherens junctions (Figure 12F). Reactome analysis also implicated the RHO GTPase cycle, Rho GTPase-IQGAP signaling, SLC2A4/GLUT4 translocation, and neutrophil degranulation (Figure 12G). These predictions nominate possible SDC4-associated programs and are not equivalent to experimental gene knockout or evidence of downstream control.

### Transcriptome-inferred metabolic pathway remodeling in AM

To examine metabolism-associated transcriptional patterns in AM, we applied scMetabolism to single-cell transcriptomic data (Supplementary Table S6). This analysis yields pathway-gene scores rather than direct metabolomic profiles.

AM epithelial cells showed higher expression-derived scores for lysine degradation, D-glutamine/D-glutamate metabolism, histidine metabolism, pantothenate/CoA biosynthesis, and fatty-acid biosynthesis, with lower scores for pyruvate metabolism, glycolysis/gluconeogenesis, and the TCA cycle (Figure 13B). Together with the measured tissue metabolite differences and crotonyl-CoA assay signal, these patterns support further investigation of metabolic-Kcr coupling in AM.

**Figure 13:**
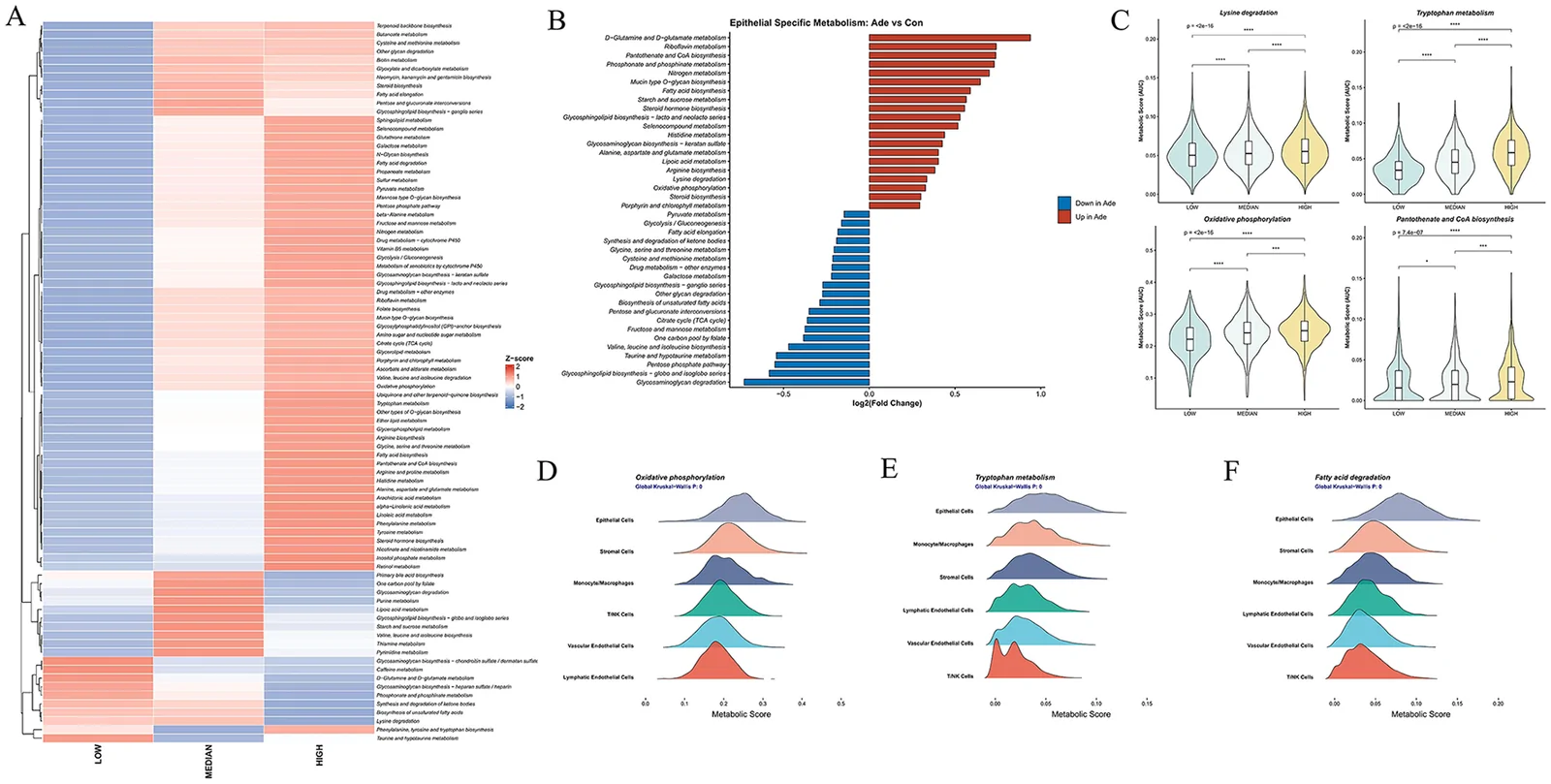
Transcriptome-inferred metabolic pathway patterns in adenomyosis. **(A)** Heatmap of expression-derived scores for 84 metabolic pathways across epithelial-cell Kcr-score groups. **(B)** Comparison of pathway-gene scores between adenomyosis (Ade) and control (Con) epithelial cells. **(C)** Lysine degradation, tryptophan metabolism, oxidative phosphorylation, and pantothenate/CoA biosynthesis scores across Kcr-score groups. **(D–F)** Corresponding pathway scores across major cell populations. These scores do not directly measure metabolite abundance or metabolic flux.

Lysine-degradation, tryptophan-metabolism, oxidative-phosphorylation, and pantothenate/CoA-biosynthesis scores increased across epithelial Kcr-score groups (Figures 13A, C). Because both sets of scores were derived from gene expression, their association does not independently establish acyl-CoA production or a causal metabolic-to-Kcr relationship.

Epithelial cells showed prominent expression-derived scores for oxidative phosphorylation, tryptophan metabolism, and fatty-acid degradation relative to other cell populations (Figures 13D–F). This localization motivated epithelial-focused interpretation, but tissue metabolite and immunoassay measurements do not identify the cellular or subcellular source of the observed signals.

Together, the transcriptional analyses nominate lysine handling and other metabolic programs for mechanistic investigation alongside the tissue biochemical observations.

## Discussion

This study integrates clinical, metabolomic, biochemical, and transcriptomic evidence to characterize a metabolic and crotonylation-related epithelial landscape in adenomyosis. Rather than indicating a broad systemic metabolic abnormality, the adjusted clinical analyses shifted the biological emphasis toward local tissue remodeling. Public endometrial metabolomics demonstrated alterations in lysine-related metabolites, carnitine, and multiple lipid species, while the newly measured central-metabolism panel identified higher glutamate and AMP together with exploratory increases in ADP, 3-phosphoglycerate, and 2-phosphoglycerate. These findings extend the evidence beyond transcriptomic inference and indicate that AM tissue is characterized by a locally altered metabolic environment. In particular, the increases in lysine, 5-aminopentanoic acid, and 5-hydroxylysine, together with prior experimental evidence linking lysine catabolism to histone crotonylation, provide a biochemical rationale for examining Kcr in AM.

This metabolic context was accompanied by several complementary crotonylation-related observations. Crotonyl-CoA immunoassay estimates were higher in AM homogenates, and H3K18cr normalized to total H3 was also increased. Although acetyl-CoA and glutaryl-CoA were not quantifiable in the targeted panel, the measured crotonyl-CoA signal, together with the histone Kcr findings, supports the presence of altered crotonylation-related signals in AM tissue. At the transcriptional level, epithelial cells showed prominent Kcr-related scores, while expression-derived metabolic analyses implicated lysine degradation, oxidative phosphorylation, glycolysis-related pathways, and other metabolic programs. Because these pathway scores are inferred from gene expression rather than direct metabolite flux, they should not be interpreted as evidence of a defined metabolic route to crotonyl-CoA production. Nevertheless, the convergence of tissue metabolite measurements, crotonyl-CoA estimates, histone Kcr, and epithelial transcriptional patterns supports further investigation of metabolic–crotonylation coupling in AM. Direct flux analysis and substrate-manipulation experiments will be required to clarify this relationship.

A second major observation concerns the potential association among Kcr, p65, and SDC4. RELA/p65 and other NF-κB-family members were predicted as candidate regulators of SDC4, consistent with the established transcriptional activity of p65 (31). In endothelial-like EAhy926 cells, p65 binding to two NF-κB sites within the SDC4 promoter has been shown to mediate TNF-α-responsive SDC4 expression (32), providing a mechanistic precedent for investigating a similar regulatory connection in AM. Related inflammatory and matrix-remodeling processes have also been described in pelvic inflammatory disease and endometriosis, although these conditions are biologically distinct from adenomyosis (33, 34). In parallel, Pan-Kcr signal was detected in p65 immunoprecipitates and was higher in the AM specimen after normalization to recovered p65. These data do not establish direct crotonylation of a specific p65 lysine residue, but they provide a complementary p65-associated Kcr observation that warrants further study.

The histone and p65-associated findings also raise two non-exclusive possibilities for how crotonylation might interface with SDC4-related epithelial programs. Histone crotonylation may influence transcriptional permissiveness, consistent with studies of AF9 YEATS-domain recognition and ACSS2-related regulation of Kcr in other biological systems (35–37). Alternatively, crotonylation within p65-containing protein complexes may influence transcriptional signaling. The present tissue assays cannot distinguish between these possibilities: H3K18cr was not localized to the SDC4 promoter, and the p65 immunoprecipitation experiment did not identify a site-specific p65 modification. Chromatin-occupancy assays, site-specific proteomics, and functional perturbation will therefore be necessary to determine whether either mechanism contributes directly to SDC4 regulation in AM.

SDC4 emerged as the most consistently prioritized epithelial candidate in the integrative analysis. Its expression was enriched in epithelial cells with high Kcr-related transcriptional scores, and SDC4-positive cells were preferentially distributed within a less differentiated region of the inferred trajectory. This pattern is compatible with epithelial plasticity but should not be interpreted as evidence of lineage ancestry or a fixed disease stage. The known biology of SDC4 nevertheless provides a plausible framework for the associated transcriptional programs. SDC4 participates in extracellular-matrix organization, PKC-α and phosphoinositide signaling, Rho-family pathways, and cell-matrix adhesion (28, 38–40), while previous studies have also linked SDC4 to TGF-β signaling and receptor shedding (41, 42). Consistent with these functions, *in silico* perturbation and enrichment analyses associated the SDC4-centered network with epithelial adhesion, junctional organization, cytoskeletal regulation, and differentiation-related genes such as ATOH8 (43). The predicted RHO-GTPase programs and representative P-FAK staining further support an adhesion- and mechanosignaling-related interpretation, in line with reported effects of SDC4 on FAK phosphorylation and cell mechanics (44, 45). Similarly, the accompanying P-Smad3 staining provides a fibrosis-related tissue observation that can be considered in the context of reported SDC4–TGF-β feedback and matrix-stiffness-dependent Smad responses (41, 46). These associations are particularly relevant to AM, where disruption of epithelial junctions, polarity, and epithelial-mesenchymal plasticity may contribute to abnormal tissue organization and invasion (1, 47).

The broader regulatory context of SDC4 may also extend beyond cell-autonomous epithelial programs. CellChat predicted distinct communication patterns between SDC4-positive epithelial cells and stromal, endothelial, and immune populations, suggesting that the SDC4-associated epithelial state may participate in broader microenvironmental crosstalk. Wnt signaling and mechano-chemical interactions described in other fibrotic and inflammatory tissues provide relevant context for these predicted communication patterns (48, 49). In addition, FoxO-related and GLUT4-translocation terms identified after computational SDC4 perturbation suggest a potential interface between adhesion signaling and nutrient-responsive pathways, consistent with established roles of FOXO factors and glucose transporters in reproductive tissues and endometriosis (50–53). WFDC2 was retained as a secondary epithelial candidate, supported by previous reports of elevated serum HE4 in adenomyosis (54), but the more consistent data-driven prioritization of SDC4 motivated the present mechanistic focus.

Taken together, these findings define a testable framework in which local metabolic remodeling and crotonylation-related changes are associated with an SDC4-enriched epithelial state in adenomyosis, with p65 representing a candidate molecule for investigating potential connections among these observations. The current data do not establish a linear metabolic–Kcr–p65–SDC4 pathway; rather, they identify a set of convergent biochemical, transcriptional, and protein-level observations that can guide subsequent mechanistic studies. Future work should determine whether manipulation of crotonyl-CoA availability or specific histone or non-histone Kcr events alters p65 activity, SDC4 regulation, and epithelial adhesion, plasticity, or invasive behavior in AM.

### Limitations

Several limitations should be considered. Some of the biochemical validation experiments were conducted in relatively small tissue cohorts. Conventional Western blot analyses included three specimens per group, H3K18cr measurements included three specimens per group, and the crotonyl-CoA immunoassay included seven specimens per group. These sample sizes limit statistical precision, the ability to capture interindividual heterogeneity, and the generalizability of the observed differences. The p65-associated Kcr comparison was further limited to a single IP measurement per group and therefore represents only a preliminary descriptive observation without patient-level inferential statistical support. In addition, acetyl-CoA and glutaryl-CoA were not quantifiable in the targeted panel. Accordingly, these biochemical findings should be regarded as exploratory and require replication in larger, independent cohorts.

The single-cell comparisons and computational perturbations provide exploratory biological hypotheses. Functional intervention, site-specific Kcr mapping, chromatin-occupancy experiments, primary or immortalized AM epithelial models, and an appropriate animal model remain priorities for follow-up work.

Although the H3K18cr findings and the preliminary p65-associated Kcr observation warrant further investigation, their functional significance remains uncertain and requires validation *in vitro* and *in vivo*.

## Conclusion

Our integrated metabolomic, biochemical, and transcriptomic analyses delineate a local metabolic and crotonylation-related remodeling landscape in adenomyosis. These alterations converge on a distinct epithelial transcriptional state characterized by dysregulated adhesion, junctional organization, and intercellular signaling, with SDC4 emerging as a consistently prioritized epithelial feature. The accompanying H3K18cr and p65-associated Kcr signals further provide a molecular context for investigating how metabolic remodeling may interface with epithelial regulatory programs in AM. Collectively, these findings support an associative framework connecting local metabolic remodeling, crotonylation-related changes, and SDC4-associated epithelial remodeling. The proposed Kcr-p65-SDC4 association model requires validation in independent cohorts and dedicated functional studies before any causal or mechanistic relationship can be established.

## Data Availability

The original contributions presented in the study are included in the article/Supplementary material, further inquiries can be directed to the corresponding author.
