# Supplementary material for: Integrated metabolomic and single-cell analyses reveal crotonylation-related epithelial remodeling and SDC4 dysregulation in adenomyosis
Source: Front Nutr. 2026 Oct 12;13:1917718. doi: 10.3389/fnut.2026.1917718 (PMC13696502; doi:10.3389/fnut.2026.1917718)
Supplement: Supplementary file 1 [file Data_Sheet_1.pdf]

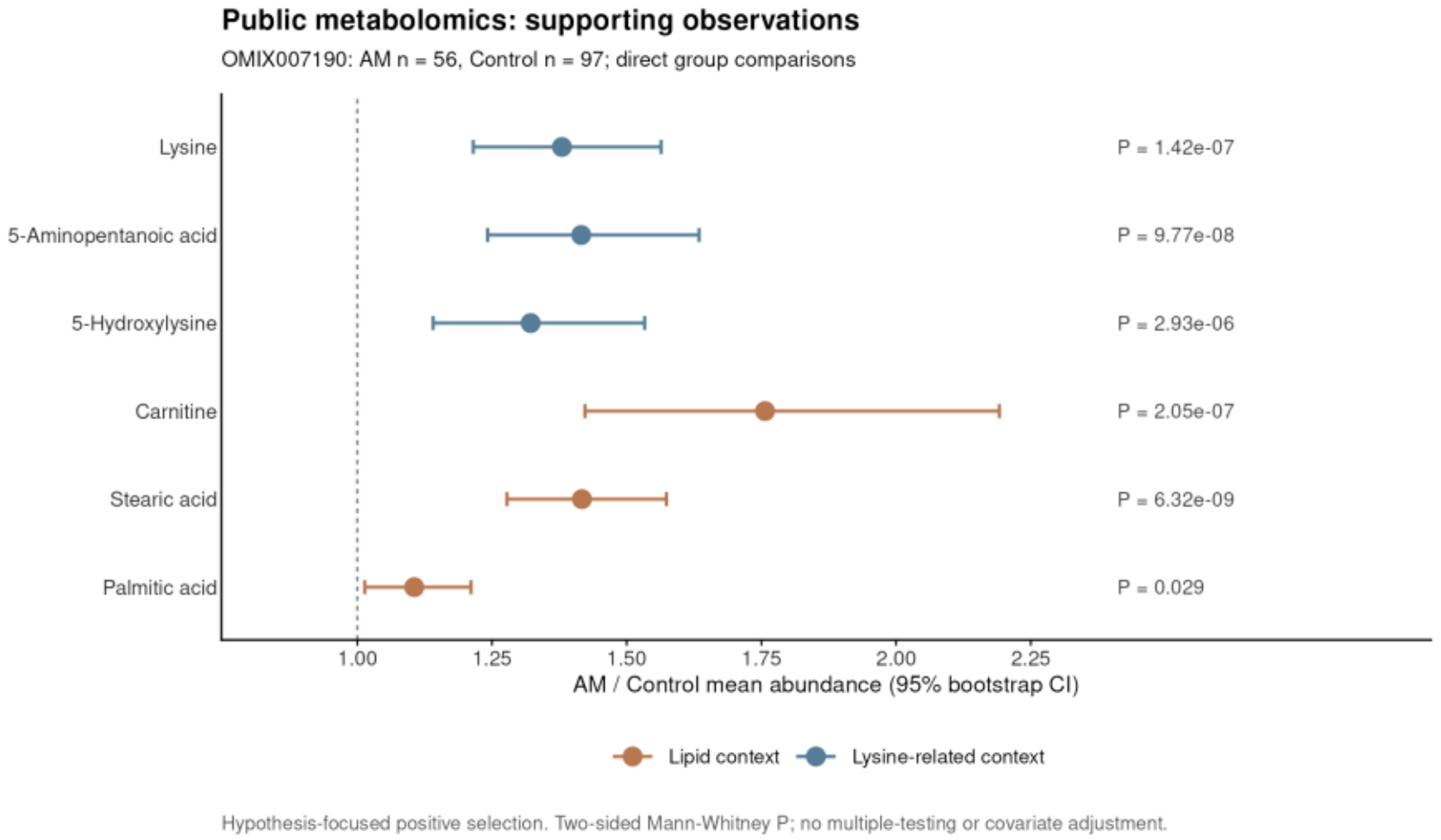

B

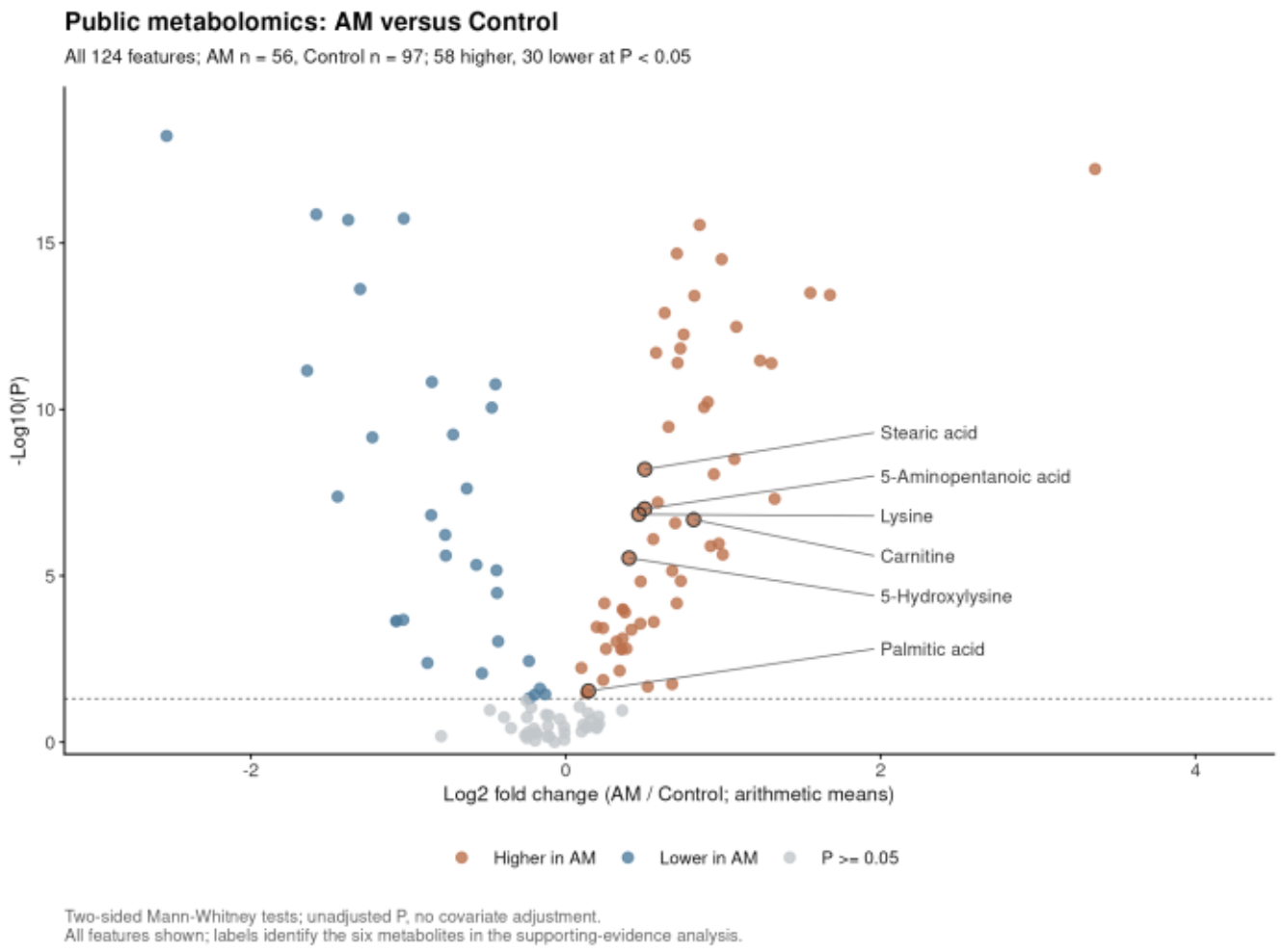

C

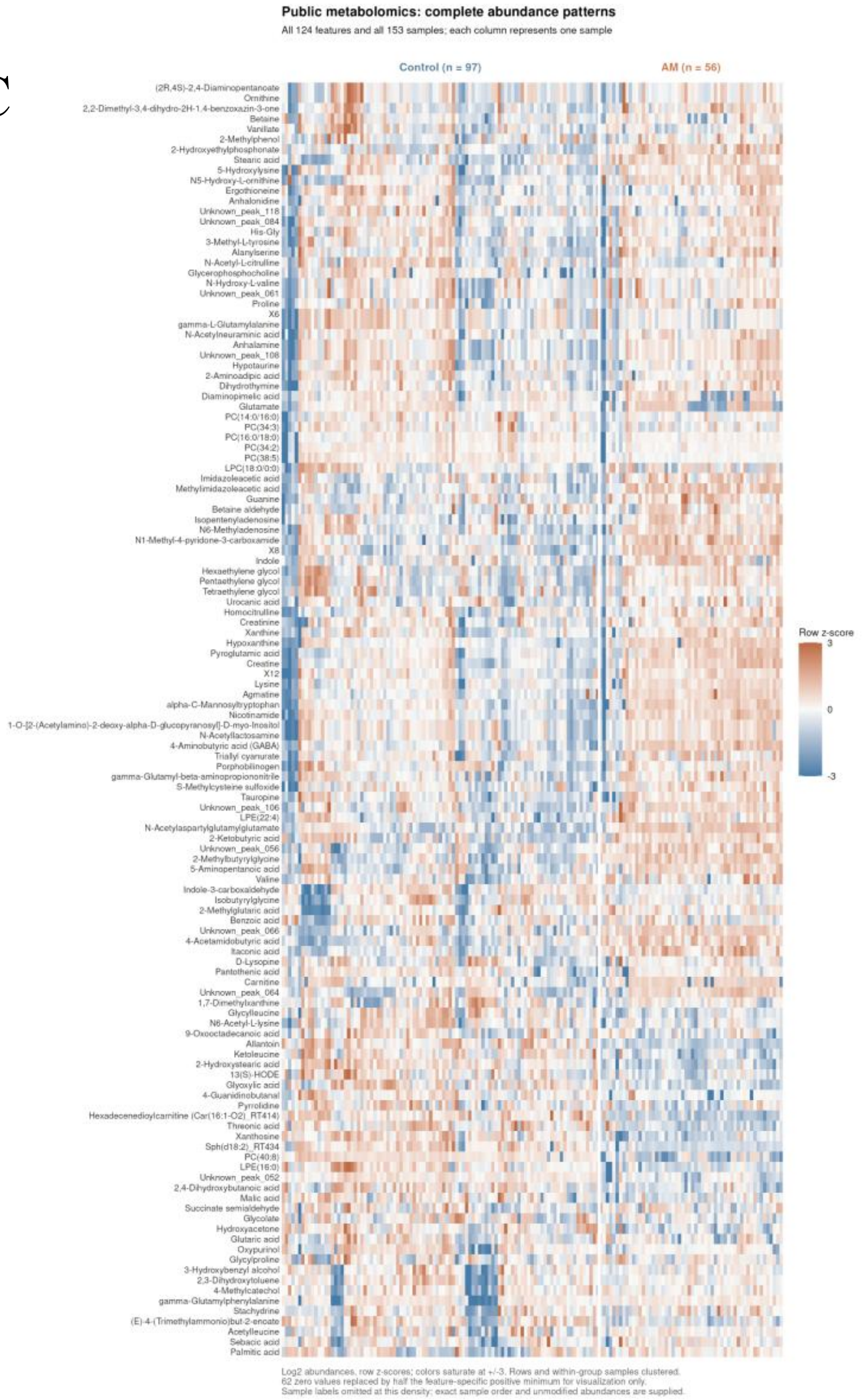

Public OMIX007190 endometrial metabolomics.
The panels show lysine-related and lipid-related metabolite differences in 56 AM samples and 97 healthy controls.
These tissue abundance changes provide metabolic context for the Crotonyl-CoA and Kcr observations.

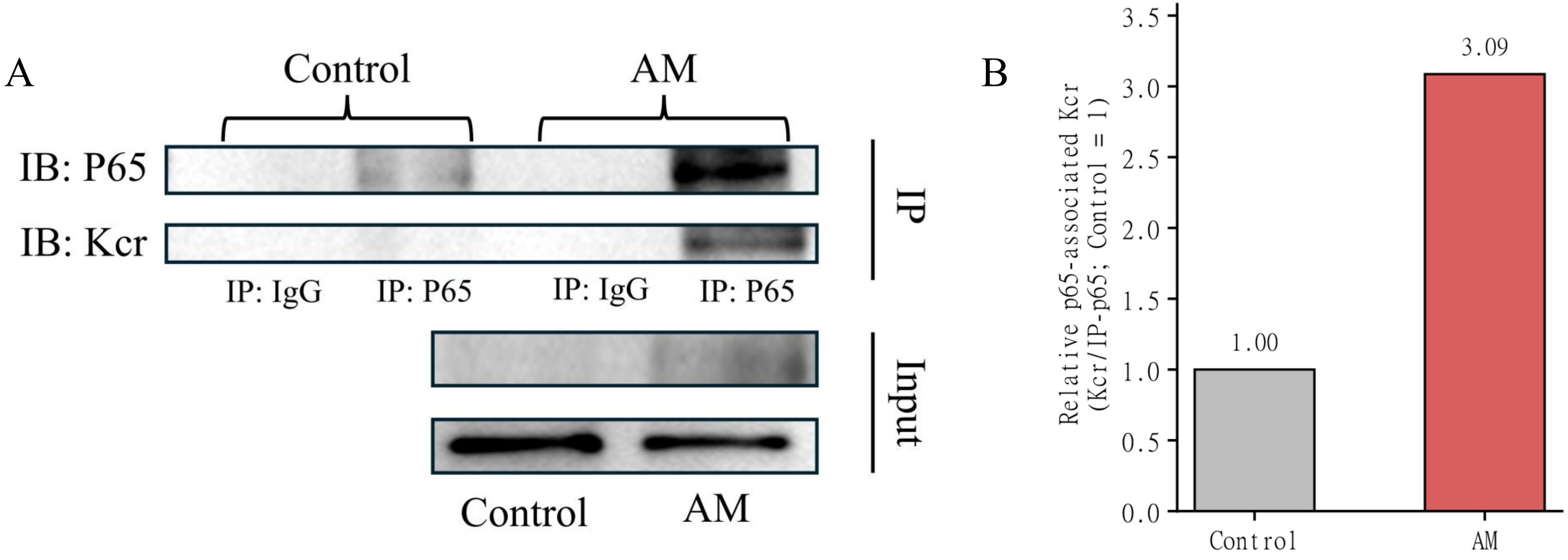

p65 immunoprecipitation and associated Kcr signal. p65 was immunoprecipitated from tissue lysates and the recovered material was probed for Pan-Kcr and p65. The Pan-Kcr signal normalized to recovered IP-p65 was higher in the AM specimen than in the control specimen. One IP measurement was obtained per group, so the comparison is descriptive and is shown without a P value or error bars. Uncropped films are retained outside the submission-ready folder.

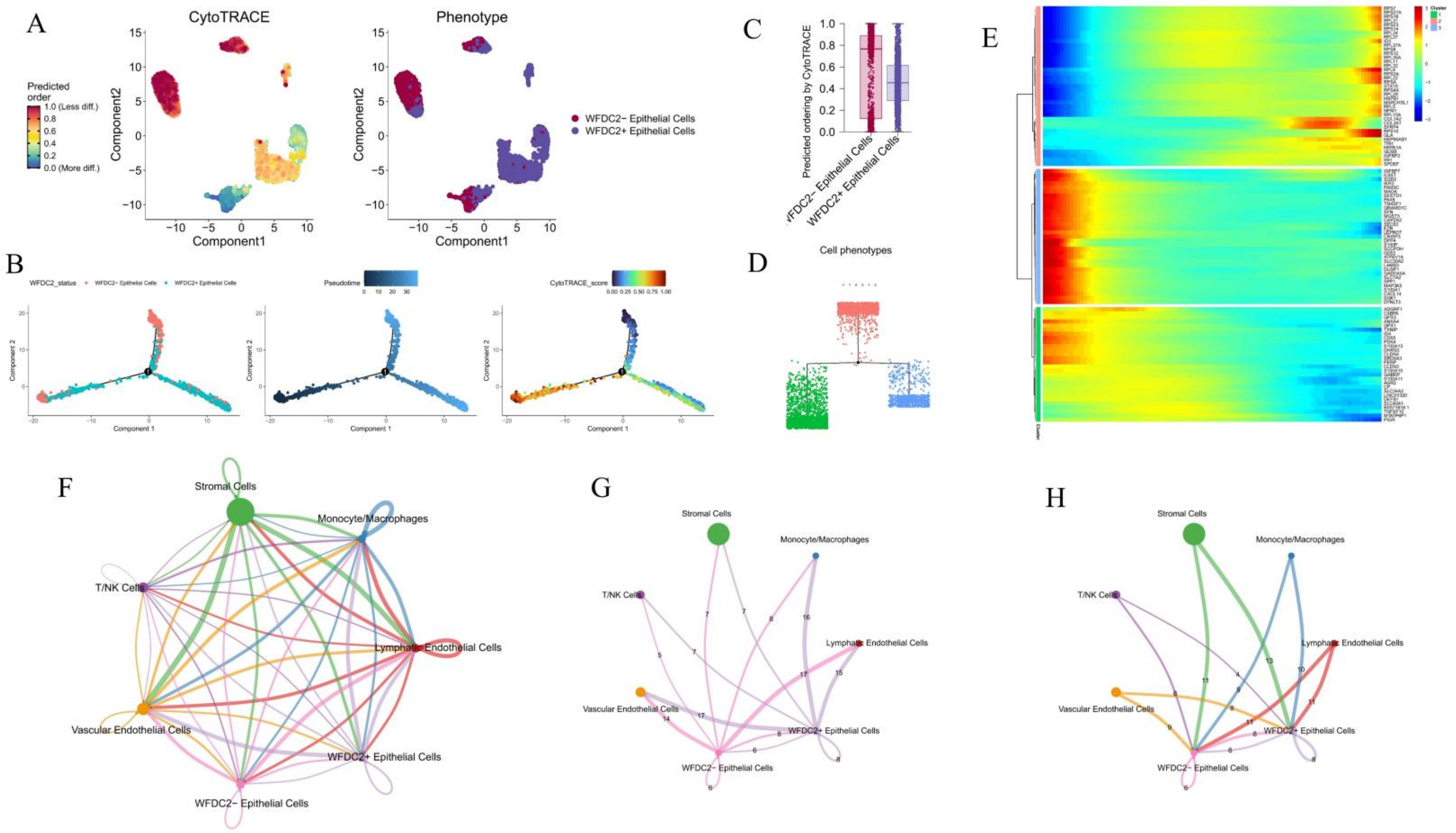

Differentiation trajectory and intercellular communication analysis of WFDC2+ epithelial cells and WFDC2- epithelial cells. (A, B) CytoTRACE-inferred differentiation scores and cell mapping of WFDC2+ and WFDC2- epithelial cells. (C, D) Monocle pseudotime trajectory and distribution of WFDC2+/- epithelial subclusters along the inferred transcriptional trajectory from Cluster 1 to Clusters 2 and 3. (E) Dynamic expression heatmap of functional gene modules (Cluster 1, 2, and 3) along the pseudotime axis, featuring genes such as ADGRF1, PAX8, SPP1, and the RPS/RPL family. (F) Global interaction strength analysis of all cell types. (G, H) Quantitative split analysis comparing outgoing (G) and incoming (H) signaling intensities between WFDC2+ and WFDC2- epithelial subclusters and other microenvironmental cell types.

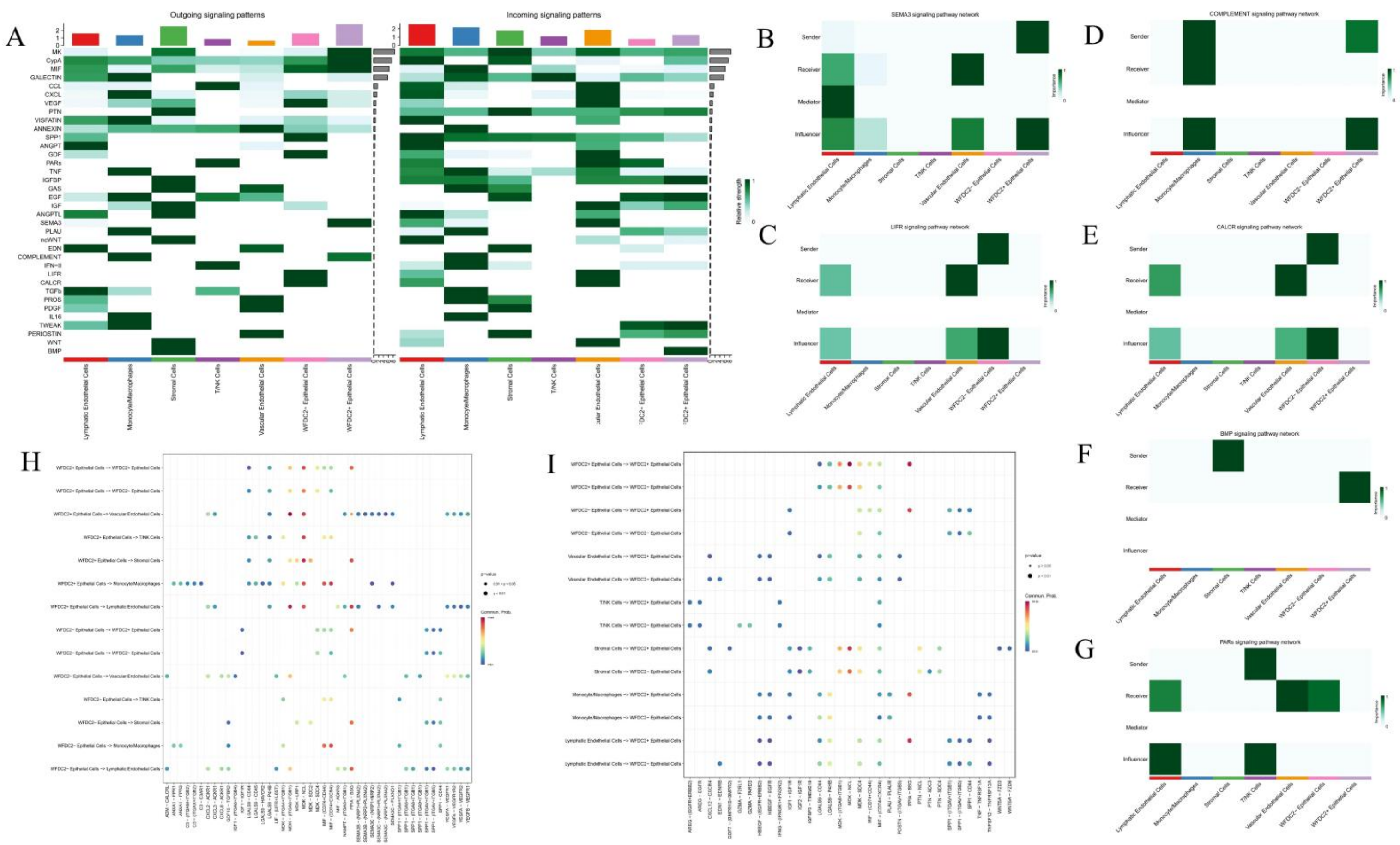

CellChat-based inference of signaling patterns and ligand-receptor interactions for WFDC2 epithelial subclusters. (A) Heatmaps of outgoing and incoming signaling patterns across various pathways. (B–G) Heatmaps displaying network centrality measures including sender, receiver, mediator, and influencer roles for cell populations within specific signaling pathways: SEMA3 (B), LIFR (C), COMPLEMENT (D), CALCR (E), BMP (F), and PARs (G). (H) Dot plot of specific ligand-receptor pairs illustrating outgoing signaling interactions from WFDC2+ and WFDC2- epithelial cells to other microenvironmental components. (I) Dot plot of specific ligand-receptor pairs depicting incoming signals received by WFDC2+ and WFDC2- epithelial cells from other cell populations.
