# Supplementary material for: Integrated metabolomic and single-cell analyses reveal crotonylation-related epithelial remodeling and SDC4 dysregulation in adenomyosis
Source: Front Nutr. 2026 Oct 12;13:1917718. doi: 10.3389/fnut.2026.1917718 (PMC13696502; doi:10.3389/fnut.2026.1917718)

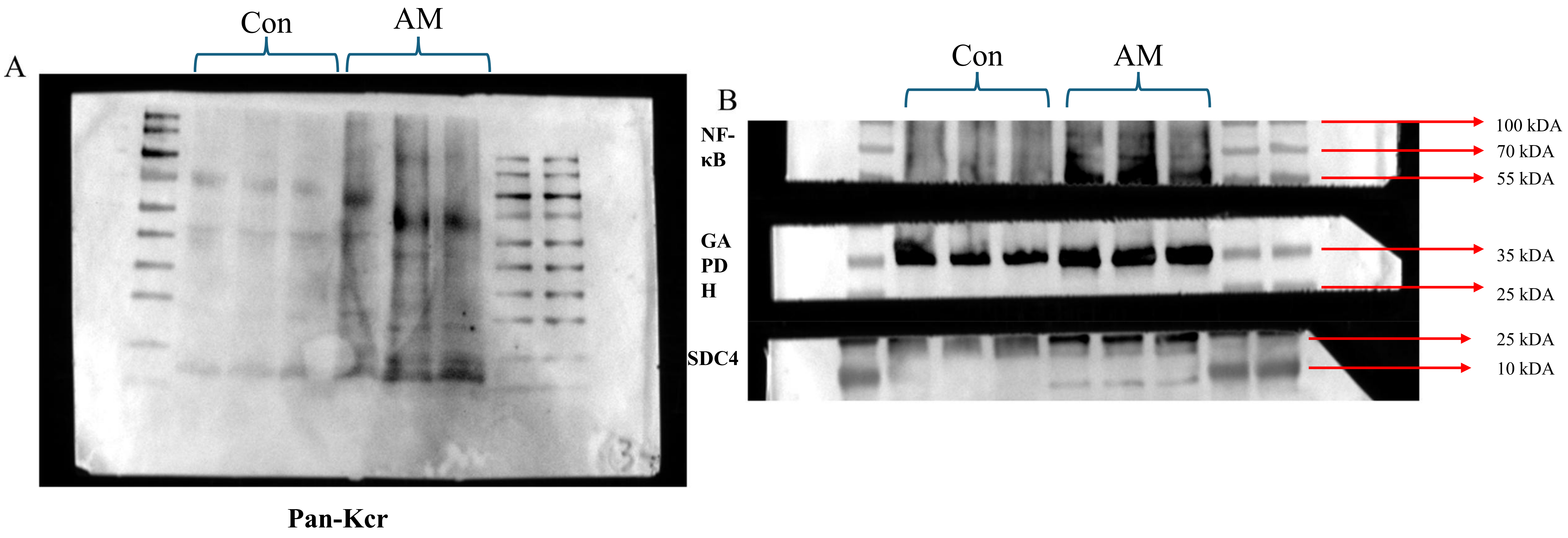
A
Con
AM
Pan-Kcr
B
Con
AM
NF-κB
GAPDH
SDC4
100 kDA
70 kDA
55 kDA
35 kDA
25 kDA
25 kDA
10 kDA

IP：P65 IB：P65

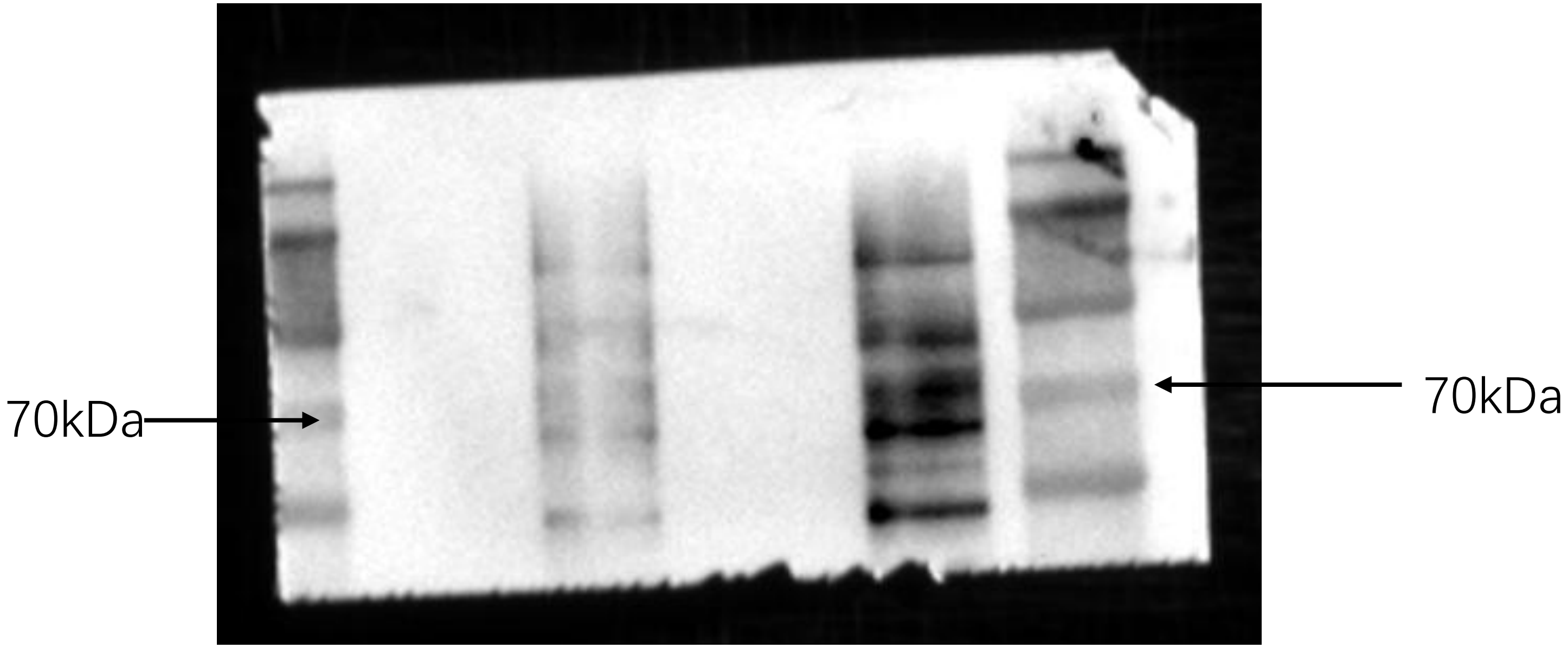

IP：P65 IB：Kcr

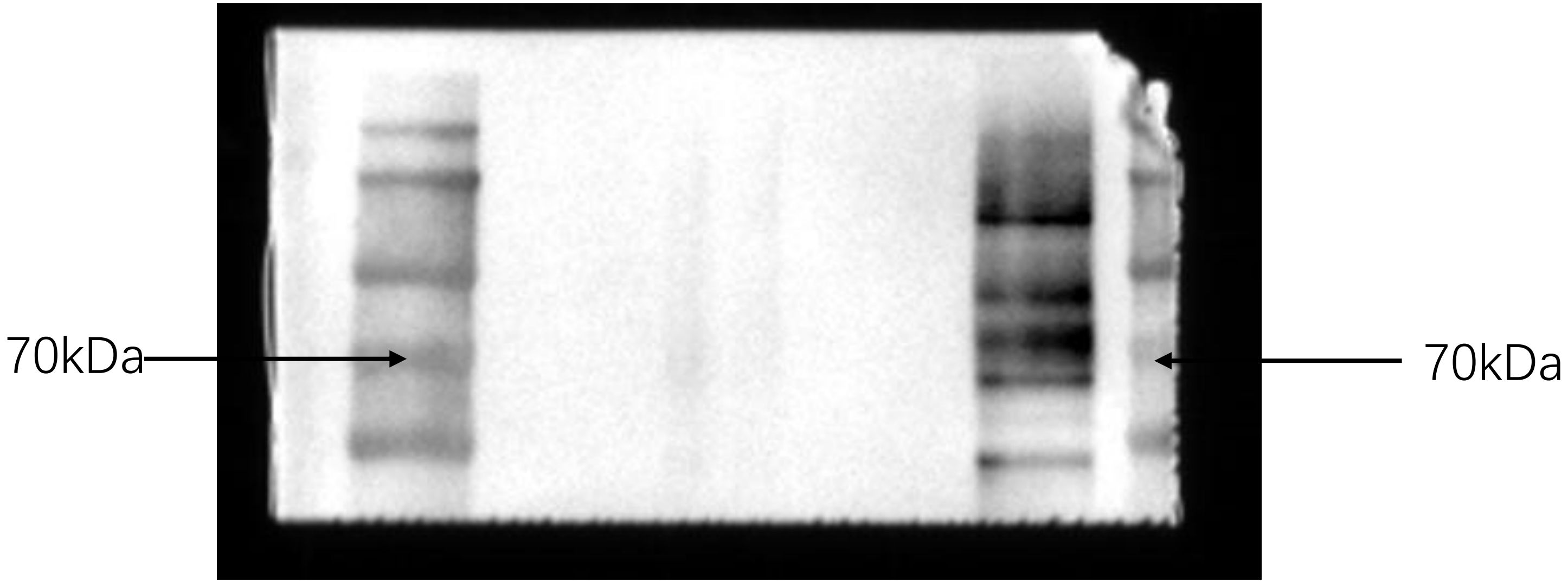

IgG-Control | Control | IgG-AM | AM

Input GAPDH

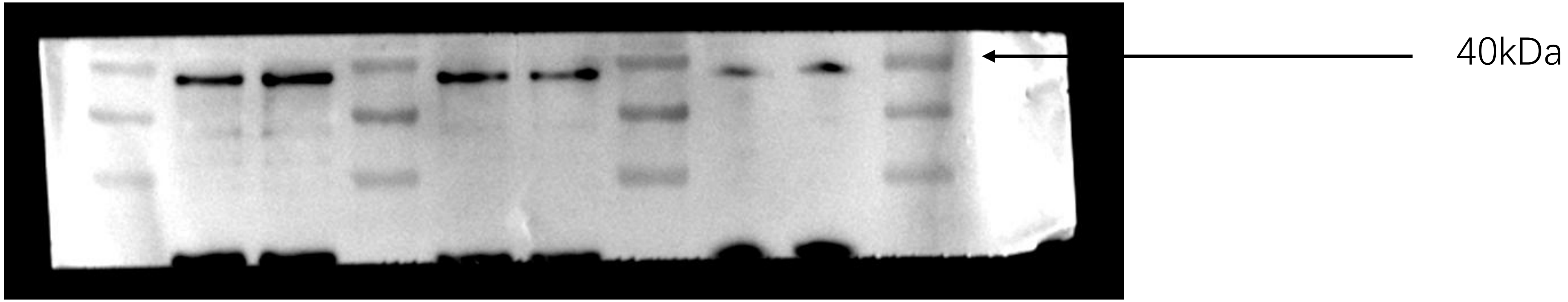

Marker | AM | Con | marker | AM | Con | marker | AM | Con | marker

Input P65

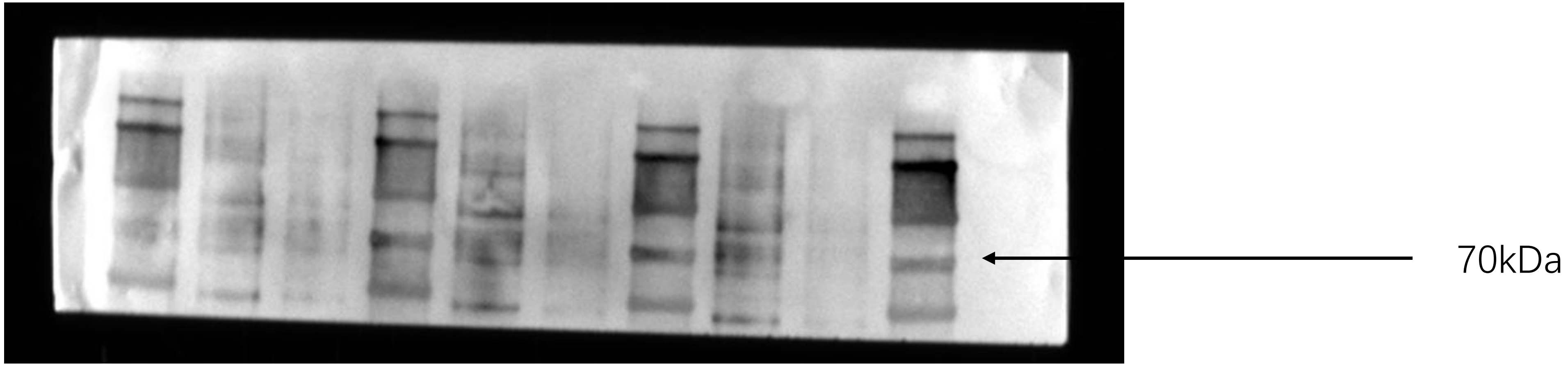

Marker | AM | Con | marker | AM | Con | marker | AM | Con | marker

H3K18cr

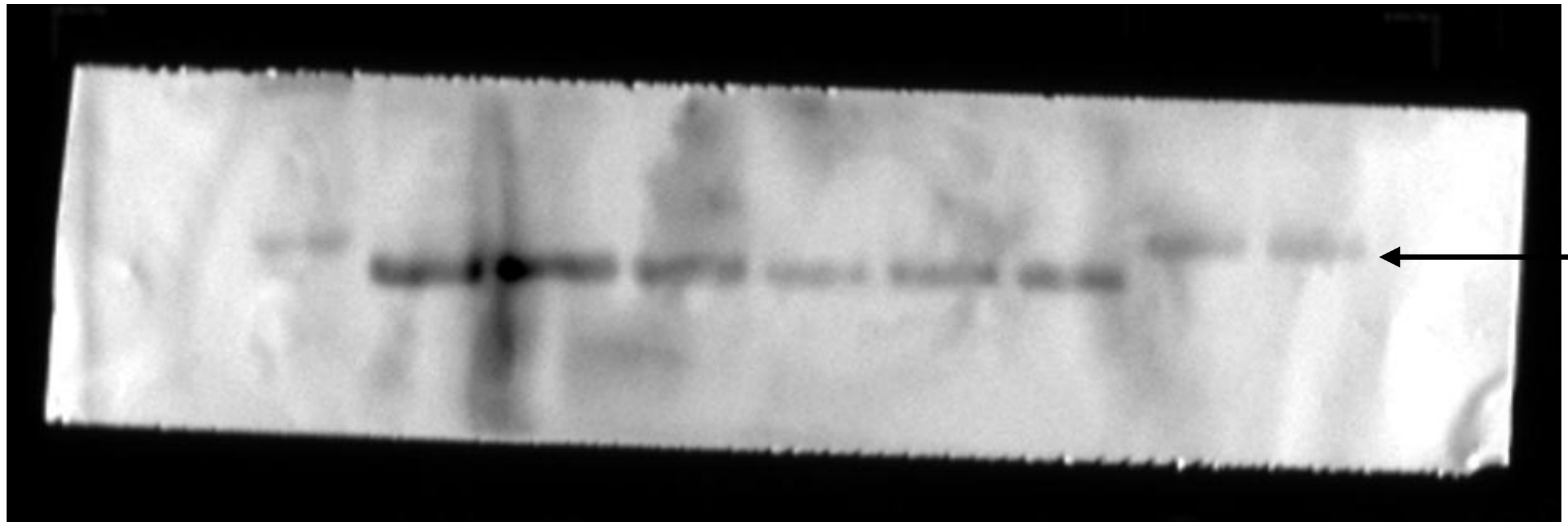

15kDa

Marker | AM | AM | AM| Con | Con | Con | Marker | Marker

Histone3

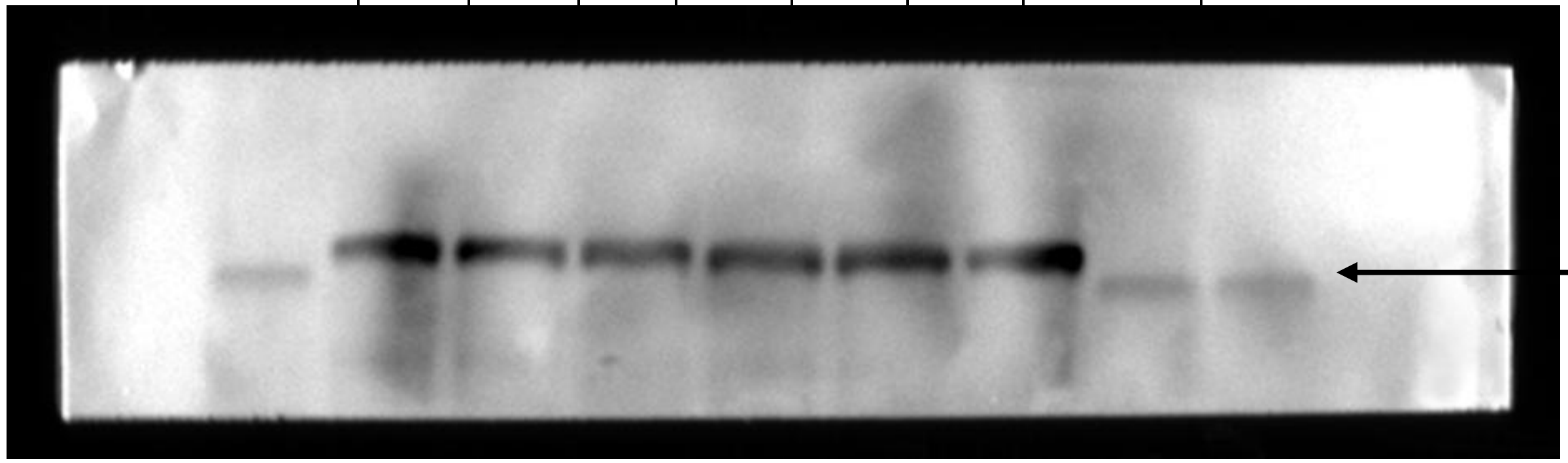

15kDa

Marker | AM | AM | AM| Con | Con | Con | Marker | Marker

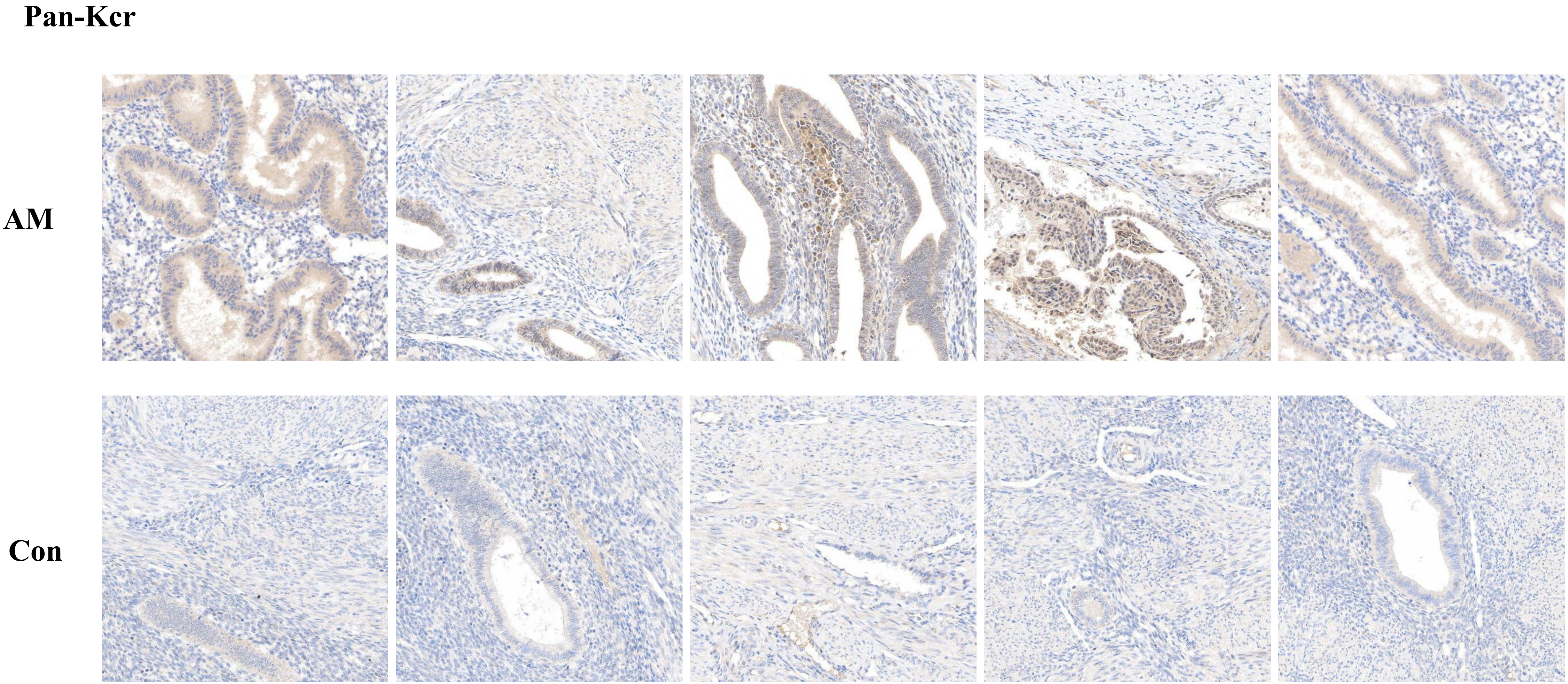
Pan-Kcr
AM
Con

P-FAK

AM

Con

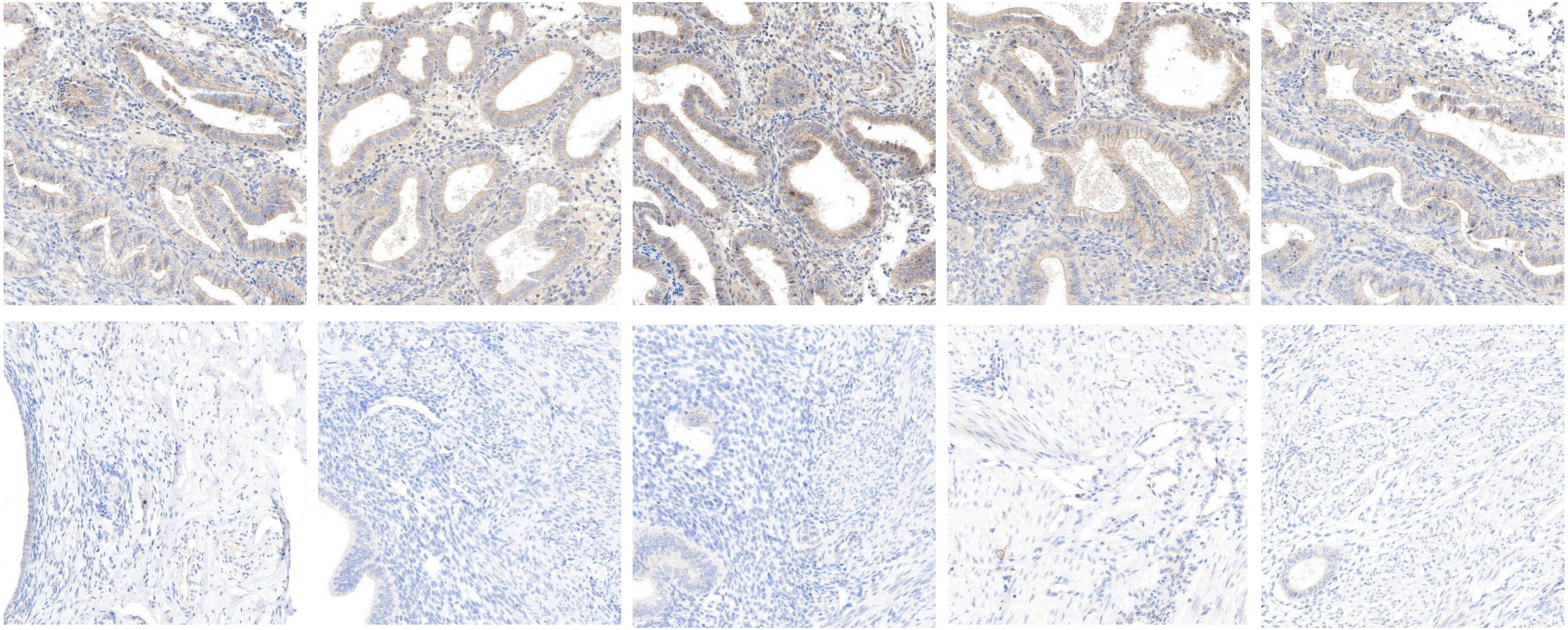

NF-κB P65

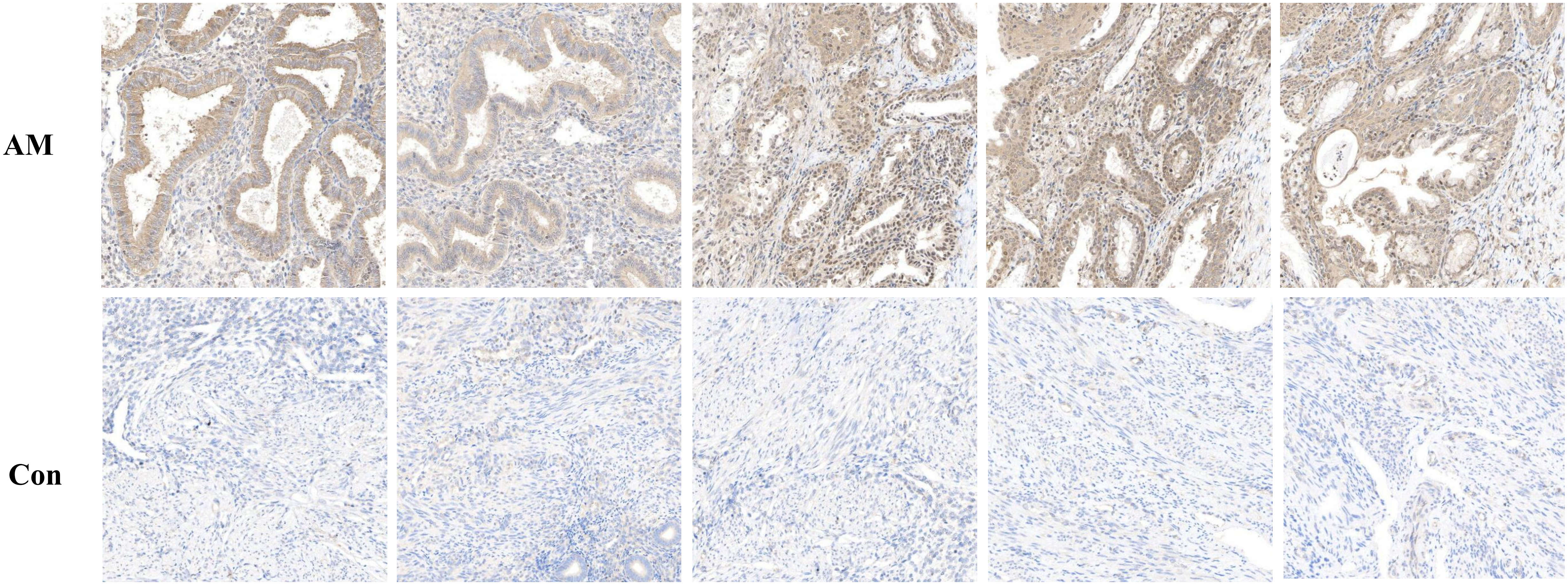

SDC4

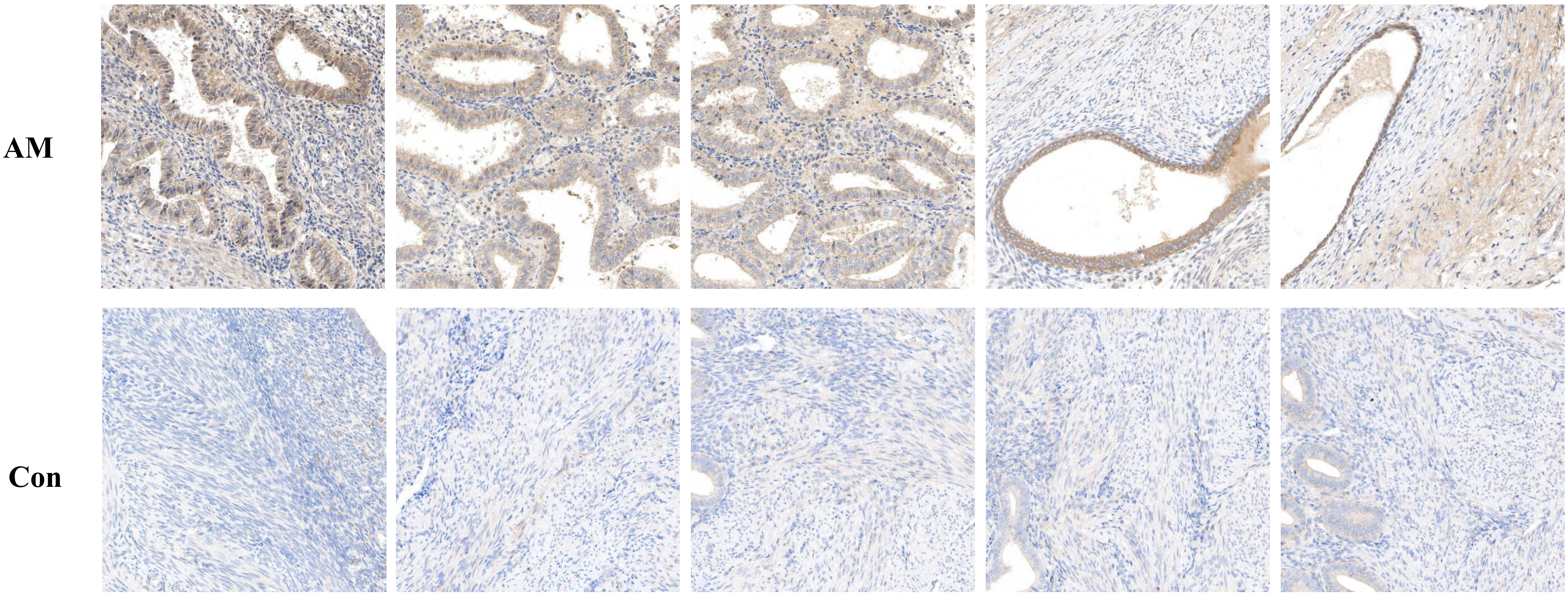

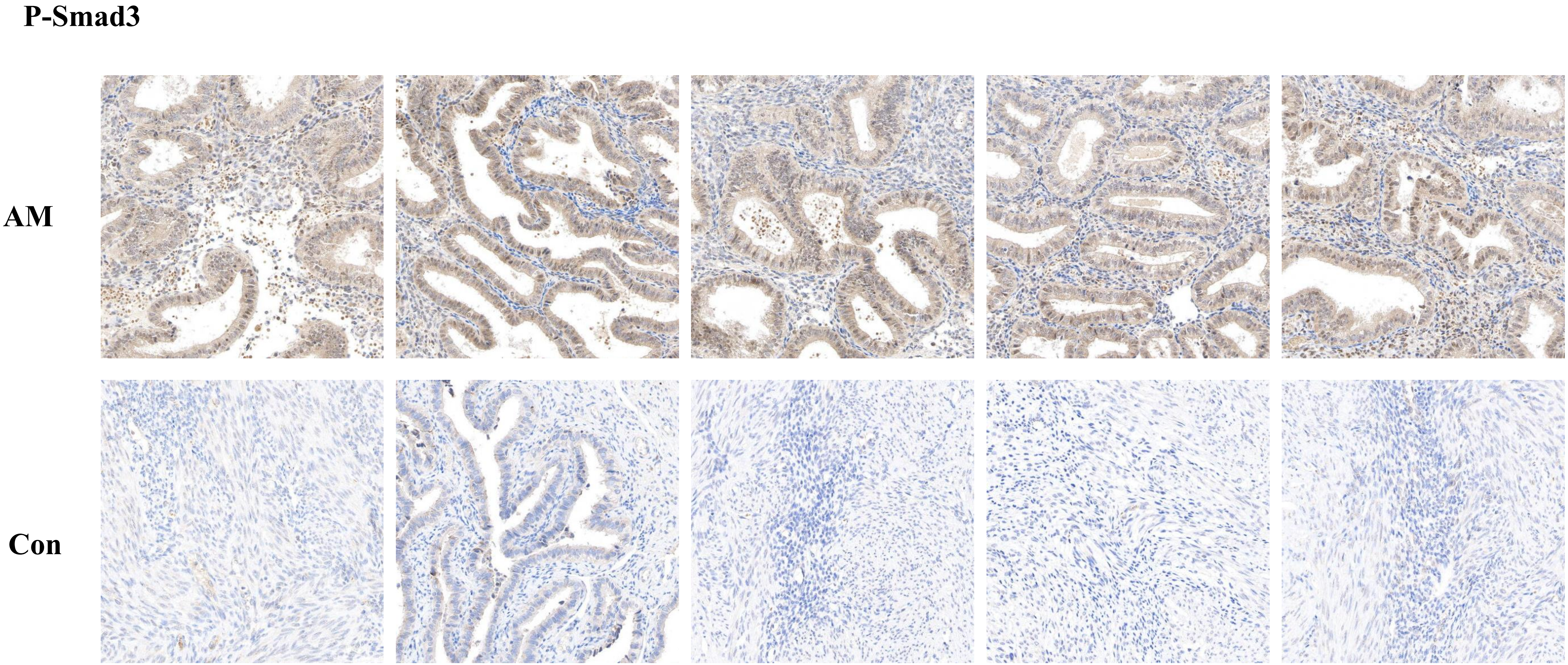
P-Smad3
AM
Con

WFDC2

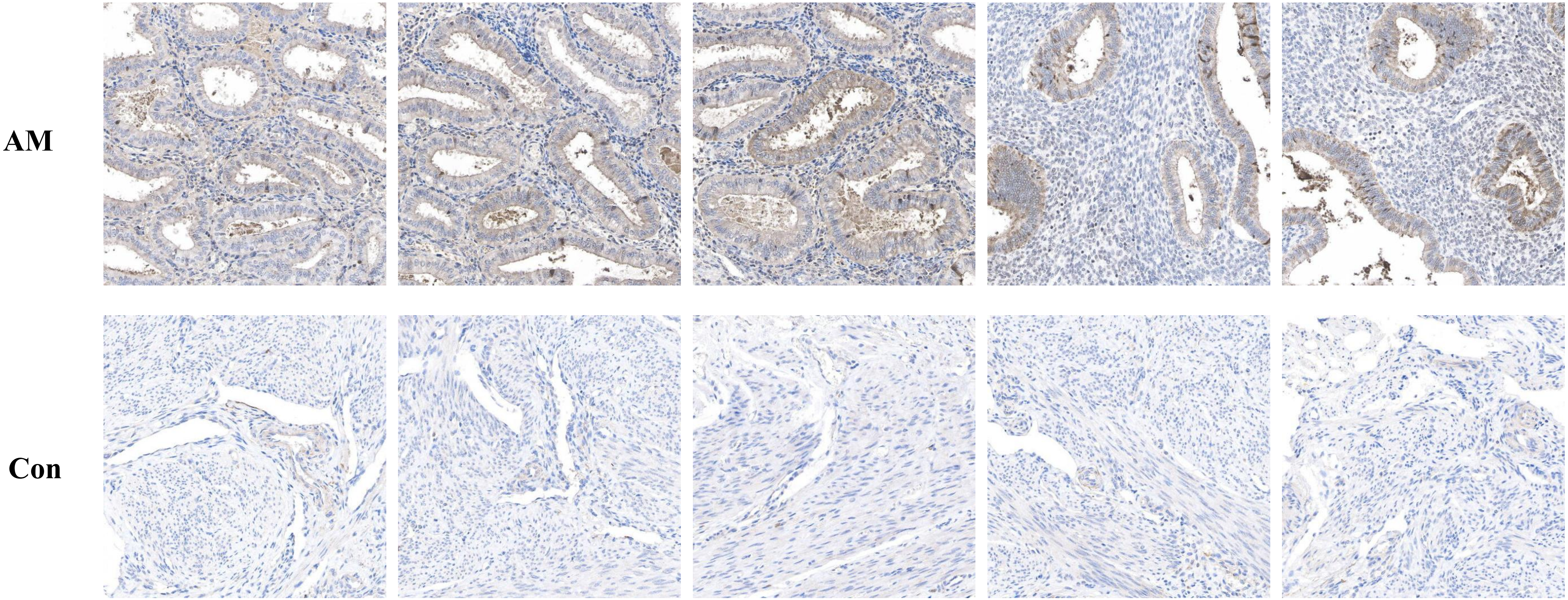

Supplement: Supplementary file 2 [file Data_Sheet_2.pdf]
